# Complexes of Fat-Soluble Vitamins with Cyclodextrins

**DOI:** 10.3390/ijms26136110

**Published:** 2025-06-25

**Authors:** Monika Zielińska-Pisklak, Konrad Adam Michalik, Łukasz Szeleszczuk

**Affiliations:** 1 Department of Pharmaceutical Chemistry and Biomaterials, Faculty of Pharmacy, Medical University of Warsaw, 1 Banacha Str., 02-093 Warsaw, Poland; 2 Department of Organic and Physical Chemistry, Medical University of Warsaw, 1 Banacha Str., 02-097 Warsaw, Poland; s092586@student.wum.edu.pl (K.A.M.); lukasz.szeleszczuk@wum.edu.pl (Ł.S.)

**Keywords:** cyclodextrin, inclusion complex, host–guest complexes, vitamins, fat-soluble vitamins

## Abstract

Vitamins are chemical compounds, or a group of closely related compounds known as vitamers, which are crucial for an organism’s metabolic functions. Vitamins are categorized as either water-soluble or fat-soluble, with this second group composed of vitamins A, D, E, and K. The low aqueous solubility of these compounds often necessitates the use of pharmaceutical excipients to benefit from their medicinal efficiency. A successful example of this is the formation of the inclusion complexes with cyclodextrins (CDs), a group of cyclic oligosaccharides, composed of glucose subunits forming a macrocyclic ring. CD complexes with fat-soluble vitamins have been consistently utilized to accomplish diverse objectives, with CDs predominantly employed as solubilizers and absorption enhancers. This article examines studies detailing the synthesis and the biological, physicochemical, and structural characteristics of the inclusion complexes formed between fat-soluble vitamins and different cyclodextrins. This research demonstrates that although the fat-soluble vitamins form stable complexes with various CDs, the kind of CDs employed significantly influences the resultant properties of the complex formed.

## 1. Introduction

The utilization of cyclodextrins (CDs) in pharmacy and medicine is widely recognized and firmly established. Numerous studies have been reported regarding the advantageous qualities of CDs, including their functions as drug delivery systems, solubilizers and absorption enhancers, agents that enhance drug stability, mask the unpleasant taste, or even act as active pharmaceutical ingredients (APIs). In addition, a lot of reviews have been published on the complexes formed between CDs and various groups of APIs such as estrogens, corticosteroids, or antibiotics. To the best of our knowledge, though, despite a significant amount of original studies, no reviews have been published specifically about the complexes of CDs and fat-soluble vitamins. These compounds, due to their structural and physicochemical properties, are natural candidates for the guest molecules of such complexes, which is clearly shown in this work.

This review aims to provide a comprehensive overview of the current state of knowledge on CD/fat-soluble vitamin inclusion complexes, focusing on the various types of CDs used, the physicochemical characterization of the complexes, and their applications. To facilitate the reception of this work, rather than dividing the analysis of the articles into sections that explain different characteristics of the complexes, we chose to write distinct paragraphs that concentrate on the specific vitamins (A, D, E, K). As a result, every paragraph in this review’s main body is structured similarly, providing comprehensive details on the procedures used to synthesize and analyze the CD-based complexes containing a specific vitamin molecule. This main part is preceded by a concise description of the most important aspects of the guest (fat-soluble vitamins) and host (CDs) molecules, presented to provide a better overview.

## 2. Vitamins

Vitamins are substances that are crucial for an organism’s metabolic functions at a minimal level, as they cannot be generated in adequate quantities by the body, thus, they must be acquired through the diet. The majority of vitamins consist of groups of related compounds known as vitamers, rather than being singular entities; for example, there are eight vitamers of vitamin E: four tocopherols and four tocotrienols. Prominent health groups enumerate thirteen vitamins, which can be grouped as water-soluble: vitamin B1 (thiamine), vitamin B2 (riboflavin), vitamin B3 (niacin), vitamin B5 (pantothenic acid), vitamin B6 (pyridoxine), vitamin B7 (biotin), vitamin B9 (folic acid and folates), vitamin B12 (cobalamins), and vitamin C (ascorbic acid and ascorbates) and as fat-soluble: vitamin A (VA), vitamin D—calciferols (VD), vitamin E—tocopherols and tocotrienols (VE), vitamin K—phylloquinones, menaquinones, and menadiones (VK), with this second group being the object of this review.

### 2.1. Vitamin A

**Figure 1:**
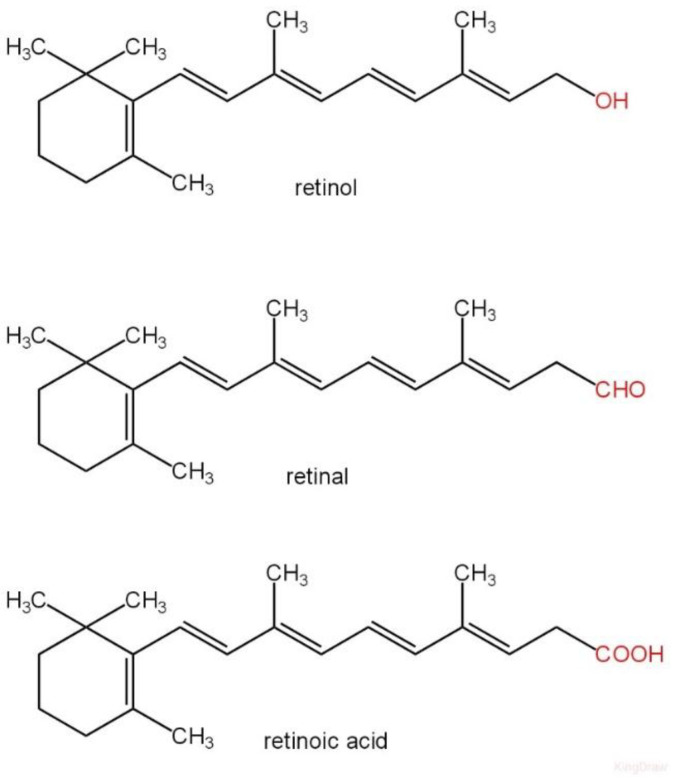
Chemical structures of vitamin A vitamers; structural differences are marked in red.

Vitamin A is a fat-soluble vitamin that serves as a vital nutrient. The designation “vitamin A” refers to a collection of chemically similar organic substances, including retinol, retinyl esters, and several provitamin carotenoids, including β-carotene. The chemical structures of the mentioned compounds are illustrated in Figure 1. VA has several functions: promoting embryonic growth, sustaining the immune system, and ensuring optimal vision. To facilitate vision, it associates with the protein opsin to create rhodopsin, the light-absorbing molecule essential for both scotopic and colour vision [1]. The recommended dietary allowance (RDA) for men and women is 900 and 700 μg of retinol activity equivalents (RAE)/day, respectively [2].

### 2.2. Vitamin D

**Figure 2:**
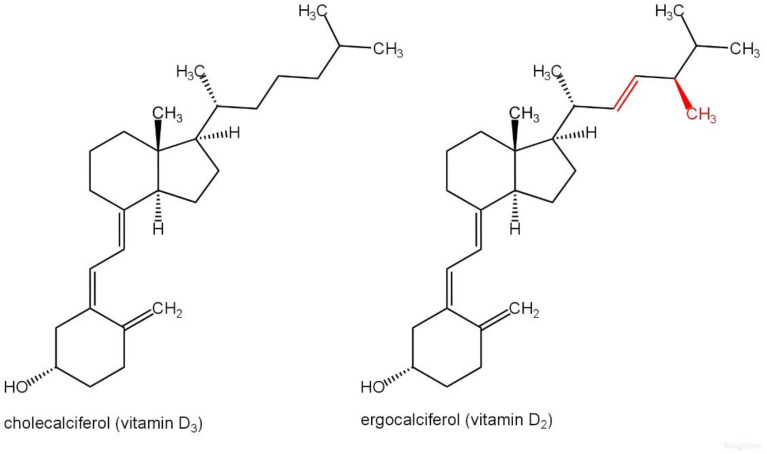
Chemical structure of vitamin D_2_ and D_3_; structural differences are marked in red.

Vitamin D comprises a class of fat-soluble secosteroids that enhance the intestinal absorption of calcium, magnesium, and phosphate, in addition to various other biological functions. Multiple vitamers of VD are present. The designation “vitamin D” pertains to either D_2_ or D_3_ or both, generally referred to as calciferol. Despite the recommendation of a chemical nomenclature for VD formed in 1981, other designations continue to be widely utilized. The different forms of VD are classified as secosteroids, indicating that one of the bonds inside the steroid rings is disrupted. The structural distinction between vitamin D_2_ (VD2) and D_3_ (VD3) resides in the side chain: vitamin D_2_ contains a double bond between carbons 22 and 23, along with a methyl group on carbon 24 (Figure 2). A variety of vitamin D analogues have been created. The chemical compositions of various VD types are presented in Table 1. The primary chemicals in this category for humans are VD3 (cholecalciferol) and VD2 (ergocalciferol).

The principal natural source of VD is the production of cholecalciferol in the deeper layers of the skin’s epidermis, initiated by a photochemical reaction with ultraviolet B (UV-B) radiation from sunshine or UV-B lamps. In addition, cholecalciferol and ergocalciferol can be acquired from dietary sources and supplements. Fatty fish are excellent providers of vitamin D, although few other foods contain it in substantial quantities. In the United States and other countries, cow’s milk and plant-based milk alternatives, along with numerous breakfast cereals, are fortified with VD [3]. Mushrooms subjected to ultraviolet light yield significant quantities of vitamin D_2_ [4]. Dietary guidelines generally presume that an individual’s VD intake is solely through oral consumption, considering the disparities in sunshine exposure across the population and the ambiguities surrounding appropriate sunlight exposure levels, especially due to the correlated risk of skin cancer [5,6]. It is assumed that the RDA of VD at the level of 20 ug is suitable, also taking into account the prevention of deficiency [7].

### 2.3. Vitamin E

**Figure 3:**
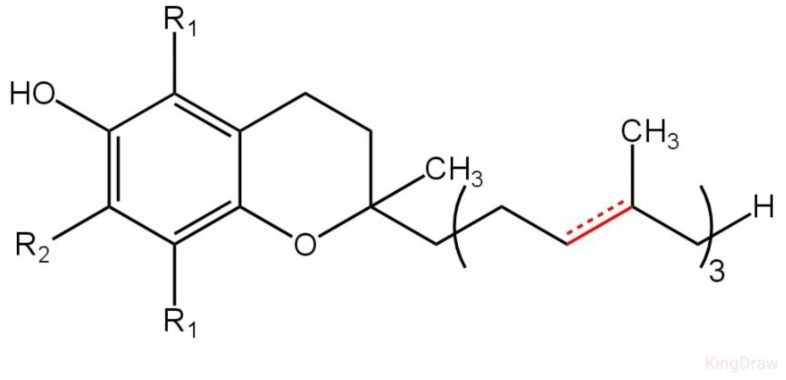
Chemical structure of substances classified as vitamin E. α-tocopherol, R_1_ = R_2_ = R_3_ = CH_3_; α-tocotrienol, R_1_ = R_2_ = R_3_ = CH_3_. β-tocopherol, R_1_ = R_3_ = CH_3_; R_2_ = H; β-tocotrienol, R_1_ = R_3_ = CH_3_; R_2_ = H. γ-tocopherol, R_1_ = R_2_ = CH_3_; R_3_ = H; γ-tocotrienol, R_1_ = R_2_ = CH_3_; R_3_ = H. δ-tocopherol, R_1_ = R_2_ = R_3_ = H; δ-tocotrienol, R_1_ = R_2_ = R_3_ = H.

Vitamin E is categorized as an essential nutrient for humans, occurring naturally in some foods and readily available as a dietary supplement. It comprises a set of eight chemical compounds, consisting of four tocopherols and four tocotrienols. VE acts as a fat-soluble antioxidant that may safeguard cell membranes from reactive oxygen species. Numerous organizations advise that individuals should ingest between 3 and 15 mg daily, but a global analysis indicated a median dietary intake of 6.2 mg per day [8,9]. Foods abundant in VE comprise seeds, nuts, seed oils, peanut butter, and vitamin E-enriched products. Symptomatic VE deficiency is uncommon and is typically attributable to an underlying issue with the digestion of dietary fats rather than a diet deficient in VE. A deficiency can lead to neurological problems. Tocopherols and tocotrienols exist in α (alpha), β (beta), γ (gamma), and δ (delta) forms, which are defined by the quantity and arrangement of methyl groups on the chromanol ring. All eight vitamers possess a chromane double ring, a hydroxyl group capable of donating a hydrogen atom to neutralize free radicals, and a hydrophobic side chain facilitating entry through the biological membranes. In tocopherols, this side chain is saturated, and in tocotrienols, it contains three double bonds. Structural differences between the tocopherol and tocotrienol forms are illustrated in Figure 3. Both natural and synthetic tocopherols and tocotrienols undergo oxidation; therefore, dietary supplements are esterified to form, i.e., tocopheryl acetates, for enhanced stability [10].

### 2.4. Vitamin K

Vitamin K consists of a collection of structurally similar, fat-soluble vitamers found in various foods and obtainable as dietary supplements. The human body requires vitamin K for the post-synthetic modification of certain proteins vital for blood coagulation and for the regulation of calcium binding in bones and other tissues. The complete synthesis involves the final modification of these proteins by the enzyme gamma-glutamyl carboxylase, which employs VK as a cofactor. The absence of VK severely disrupts blood coagulation, resulting in uncontrolled hemorrhaging. Research indicates that a deficit in VK may compromise bone integrity, potentially leading to osteoporosis, and may facilitate the hardening of arteries and other soft tissues [11].

**Figure 4:**
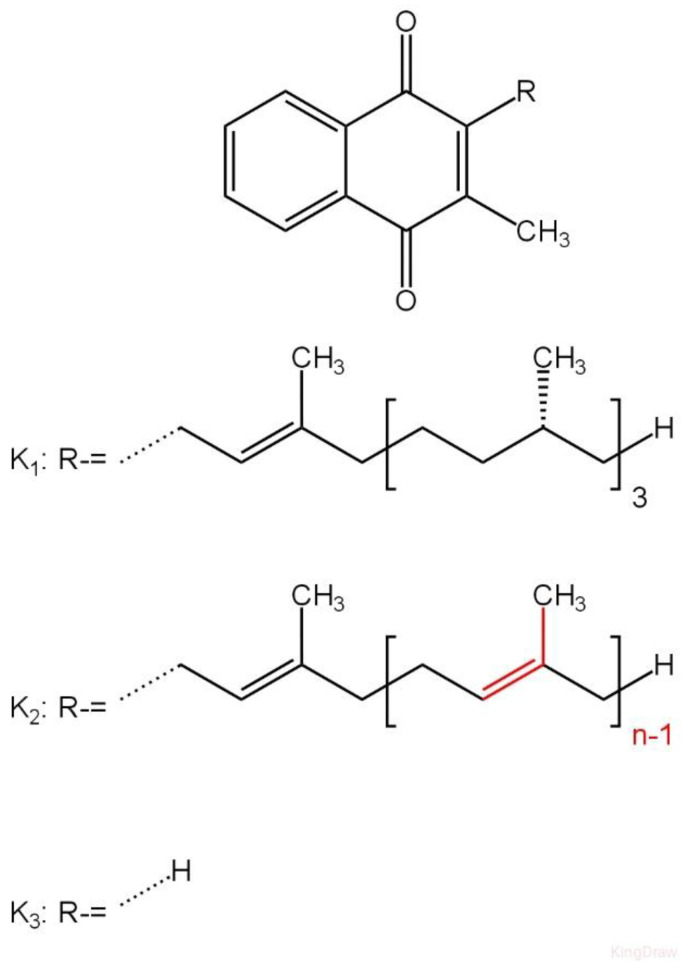
Chemical structures of K_1_ (phylloquinone), K_2_ (menaquinone), and K_3_ (menadione).

The VK family chemically consists of derivatives of 2-methyl-1,4-naphthoquinone substituted with an alkyl chain in the 3-position. VK comprises two natural vitamers: vitamin K_1_ (phylloquinone) and vitamin K_2_ (menaquinone), and one synthetic vitamer—K_3_ (menadione) (Figure 4). Vitamin K_2_ comprises several similar chemical subtypes, characterized by varying lengths of carbon side chains composed of isoprenoid groups of atoms. The two most researched forms are menaquinone-4 (MK-4) and menaquinone-7 (MK-7), composed of four and seven isoprenoid groups, respectively. Menadione is currently less frequently used in therapy due to the potential risk of causing hyperbilirubinemia and jaundice when administered parenterally. Moreover, at elevated levels, menadione seems to induce oxidative stress through its reduction to the semiquinone radical, which, in the presence of O_2_, is reoxidized to the quinone, leading to the generation of the superoxide radical anion [12,13]. It is estimated that the daily intake of VK at the level of 120 µg in men and 90 µg in women is sufficient to cover the demand [2].

## 3. Cyclodextrin Complexes with Fat-Soluble Vitamins

Cyclodextrins (CDs) are cyclic oligosaccharides consisting of six (αCD), seven (βCD), eight (γCD), or more glucopyranose units linked by α-(1,4) glucosidic bonds, synthesized by *Bacillus macerans* bacteria or obtained through the enzymatic degradation of starch [14,15,16]. Their structure resembles the shape of a truncated cone, narrower at one side and wider at the other, providing them with a special feature: due to the presence of primary and secondary hydroxyl groups, they have a hydrophilic outer surface, and due to the presence of ethereal oxygens and the carbon skeleton of glucose units—lipophilic cavity [17]. This translates into the ability to form inclusion complexes with many types of molecules, including the following: polar compounds, i.e., alcohols, acids, amines, and non-polar compounds, i.e., fat-soluble vitamins, steroid hormones, antibiotics, the volatile components of essential oils, and fatty acids [18,19,20,21,22,23,24,25]. In addition to native cyclodextrins, modified cyclodextrins, e.g., 2HPβCD, MβCD, or SBEβCD, obtained by replacing the hydrogen atoms of hydroxyl groups with various substituents, are becoming increasingly popular on the pharmaceutical market due to their improved solubility and complexing ability [26,27]. The formation of inclusion complexes with CDs serves primarily to increase the solubility in water and accelerate the dissolution rate of poorly soluble medicinal substances and to improve their bioavailability and stability [28,29,30,31]. Additionally, encapsulation by CDs is used to mask the unpleasant taste or odour, reduce the evaporation of volatile substances, protect sensitive substances from light, humidity, heat, or oxygen, reduce the phenomenon of the local irritation of the gastrointestinal mucosa, and overcome the problems of incompatibility between the drug substances [32,33,34,35,36,37,38]. Fat-soluble vitamins are regarded as good candidates for complexation by CDs due to their hydrophobic nature and appropriate molecular size that matches the CD’s cavity; thus, various interactions stabilizing this type of complex are possible, i.e., van der Waals forces, hydrophobic interactions, and hydrogen bonds [26,39,40,41].

The first publication on CDs’ inclusion complexes with fat-soluble vitamins was published by Szejtli et al. [42] in 1980. His work concerned the use of βCD to improve the stability and solubility of VD3. The PXRD, DSC, DTG, and NMR measurements confirmed that VD3 forms an inclusion complex with two βCD molecules. The molecular encapsulation by CD improved the solubility of VD3 in water and its stability at an increased temperature—VD3 was completely decomposed at a temperature of 80 °C within 24 h, while the complex with βCD retained 49% of primary activity even after 43 days [42]. Another publication by Szejtli et al. [43] proved that VK3 forms an inclusion complex with one βCD molecule. Based on UV and ^13^C NMR spectra, it was found that the benzene ring of VK3 was located in the apolar cavity of βCD, while the quinone part was located outside it. The stability of the analyzed complex was rather poor; the stability constant Kc calculated from the circular dichroism spectrum and solubility isotherm was 200 mol^−1^ [43]. Over the 40 years that have passed since the above-mentioned studies, knowledge about the inclusion complexes of fat-soluble vitamins with CDs has expanded significantly.

### 3.1. Complexes of Vitamin A and Its Derivatives with Cyclodextrins

#### 3.1.1. Preparation Methods of Inclusion Complexes with VA Derivatives

The complexes of VA and its analogues with cyclodextrins were usually prepared in a molar ratio of 1:1 or 1:2 [39,44,45,46,47,48]. However, cases of a larger number of cyclodextrin molecules per one molecule of retinol derivative can be found, i.e., 1:3 [49], 1:4 [50,51], and 1:10 [52]. The main methods for obtaining the complexes of VA and its derivatives with cyclodextrins include kneading with a small amount of solvent (water, ethanol, or their mixtures) [50,53,54,55] or without solvent [51], freeze-drying of water–ethanol [39,44,56], aqueous [52,57,58] or phosphate-buffer solution [47], electrospinning [45,59,60], and co-or nanoprecipitation [61,62,63]. The VA analogues most often formed complexes with βCD [39,46,47,48,49,54,55,62,64,65,66,67,68] or its modified derivatives, such as the following: DMβCD [48,51,52,66,68,69,70], HPβCD [41,44,50,60,61,68,71,72], TMβCD [48,68,70], SBEβCD [68], HEβCD [72], OAβCD [63], SAβCD [63], OSAβCD [67], GluβCD [73], and Chol-βCD-Ac [74]. Less frequently, other native or modified CDs, such as αCD [53,56,57,58,68], γCD [50,53,57], GalαCD [56], HPγCD [45,60,68], EγCD [44], OγCD [44], and DMγCD [70], were used.

Most often, CDs and their derivatives were used for complexation as pure substances; however, some researchers created complexes of VA derivatives with, e.g., βCD-functionalized PVA nanofibers [59], HPβCD and HPγCD nanofibrous webs [45], βCD-based nanosponges [75], magnetic chitosan βCD biopolymers [76], or a mixture of CDs with maltodextrin (MD) with different DE (dextrose equivalent) values [53].

#### 3.1.2. Methods of Analysis of Inclusion Complexes with VA Analogue

In order to explore the structures and properties of the obtained complexes of VA and its derivatives and CDs, various methods were used. The HPLC technique was applied to investigate whether VA or its derivatives undergo any chemical modifications due to their incorporation into CD [74], to determine the binding constants [52,71], to quantify the VA’s derivative content in complexes [49,51,52,61,66,71,74], to monitor the release of VA’s derivatives from complexes, to analyze whether complexation improves the stability of VA and its analogues [44,49,51,52,57,61,66,68,71,73], to assess the increase in VA skin penetration [44,52,57,74], to determine the solubility enhancement due to complexation [68,71,73], and to study photoisomerization processes [48].

The photoisomerization processes of BC, VA, its esters, retinal, and RA in the form of CD complexes, as well as their degradation under the influence of light, temperature, oxygen, etc., have often been studied using UV–Vis spectroscopy [44,47,48,53,61,72,75]. Another application of UV-Vis spectroscopy in the study of VA complexes with CDs concerned the analysis of association constants, the determination of stoichiometry [39,44,46,47,48,72], and solubility measurements [39,44,45,47,53,70]. This technique was also helpful in assessing the encapsulation efficiency and release profile [45,75].

FTIR spectroscopy was used to monitor the encapsulation process. Shifts in the bands originating from the functional groups of both the guest and host molecules provide information about the interactions occurring in the VA’s derivative/CD complex. The interaction between guest molecules and CD cavities generally leads to disappearance, attenuation, and/or shifts in the typical peaks of guest molecules [39,45,47,63,66]. Raman spectroscopy complements FTIR spectroscopy in VA’s analogues/CD complex structural studies by analyzing molecular vibrations through light scattering. The changes in the peak intensities and positions in Raman spectra indicate interactions between CD’s cavity and vitamin, such as hydrogen bonds, and provide information on molecular conformation [61,69]. The application of complementary FTIR and Raman spectroscopies helps track complex formation and allows assessing complex stability under varying conditions (e.g., light, temperature, oxygen).

The SEM and TEM techniques were utilized to investigate the size and morphology of the self-assembled architecture of the VA/CD complexes. SEM was ideal for surface morphology and topographical analysis, whereas TEM provided insights into the internal structures at atomic resolution [39,45,59,62,63,66,67,74,75,76]. In the research of Xu et al. [39], free VA acetate, βCD, and their inclusion complexes were easily distinguished by SEM observations. The surface morphology of βCD and VA acetate exhibited substantial differences; βCD’s surface was predominantly flat with minor irregularities, but the VA acetate surface had cracks. The physical mixture of the two samples did not demonstrate a consistent structural alteration. Conversely, a layered stacking structure was seen in the freeze-dried VA acetate/βCD inclusion complex sample. Therefore, the SEM micrograph could serve as evidence for the creation of an inclusion complex between VA derivatives and CDs [39].

The morphology and the fibre diameter of the βCD-functionalized PVA nanofibers loaded with VA acetate were analyzed by a field-emission SEM by Lemma et al. [59]. The result showed that the incorporation of VA acetate in the electrospun PVA/β-CD fibres had little effect upon the morphology; the average fibre diameter in the sample containing VA ester was slightly smaller compared to the control one [59]. Also, in the research conducted by other scientists, the SEM images showed that the surface morphology of the complexes with VA derivatives was totally different from the morphology of the pure substrates and physical mixtures, which confirmed the formation of the complexes. In the study of Niu et al. [67], SEM images demonstrated that the physical mixture of BC and OSβCD maintained the original morphology of the free substances, with BC crystals adhering to the surface of OSβCD. In turn, the BC/OSβCD inclusion complex exhibited a dense and uniform plate-like structure without any characteristic shapes of free BC and OSβCD, which indicated a strong interaction between the host and guest molecules. These results indicated the formation of the BC/OSβCD inclusion complex [67].

The TEM technique enables a more sophisticated analysis of the structure of complexes at the atomic level. Kim et al. [63] employed this method to investigate the supramolecular architecture of complexes formed by amphiphilic OAβCD and SAβCD (mono-oleamide or mono-stearamide modifications on the C6 of βCD) with VA. The fact that the self-assembled composites of VA/SAβCD exhibited a mean diameter of 24.1 nm, while VA/OAβCD had a higher average diameter of 42.5 nm, could be attributed to the presence of a double bond in the latter. The greater size diversity observed in the case of the VA/OAβCD particles compared to the VA/SAβCD ones could likewise be ascribed to the oleamide structure. The saturated chains in VA/SAβCD were more conducive to the formation of regular and stable assemblies. The varying building components of SAβCD and OAβCD resulted in unique self-organized nanostructures [63].

X-ray structural analysis techniques play a crucial role in characterizing the inclusion complexes of CDs. These methods provide valuable insights into the structural modifications induced by complexation, revealing the key aspects of molecular organization, crystallinity, and host–guest interactions. PXRD is widely used to confirm complex formation by comparing the diffraction patterns of pure components and complexes, detecting changes in the crystallinity and possible polymorphic transformations. In turn, SC-XRD allows one to estimate unit cell parameters and determine the exact location of the guest molecule in CD’s cavity, providing a detailed 3D structure of the host–guest system. Obtaining single crystals suitable for the X-ray diffraction studies of CD inclusion complexes with fat-soluble compounds is a significant challenge. These guest molecules often exhibit high conformational flexibility, low polarity, and poor crystallinity, which impedes the orderly packing required for single-crystal formation. Furthermore, their oily or semisolid physical state, combined with sensitivity to light and oxidation, further complicates the long and delicate crystallization process. These physicochemical properties are unfavourable for the controlled growth of large, well-defined single crystals and often lead to amorphous or polycrystalline products, making them unsuitable for SC-XRD analysis [77]. As a result, researchers studying these systems typically rely on alternative analytical methods such as PXRD or solid-state NMR, which are more applicable for partially crystalline or amorphous materials [78].

In the study of Celebioglu and co-authors, the PXRD technique confirmed the complex formation between CDs and VA acetate in CD nanofibrous webs (NWs) and proved its amorphous form. The pure VA acetate powder exhibited crystalline diffraction peaks at 9.1°, 17.3°, 18.5°, 20.1°, and 24.4°, while pure HPβCD-NWs and HPγCD-NWs had broad-halo PXRD patterns indicative of their amorphous characteristics. The crystalline peaks of VA acetate were evident in the VA acetate/CD physical mixture due to the lack of complexation phenomenon in the samples. Despite this, the VA acetate/HPβCD-NW exhibited an amorphous pattern akin to that of pure HPβCD-NWs, while the VA acetate/HPγCD-NW displayed an almost amorphous pattern with weak characteristic peaks at 17.3°, 18.5°, and 20.1° of VA acetate, suggesting that the VA acetate crystals were likely dispersed in diminutive sizes within the nanofibrous matrix [45]. A similar application of PXRD can be found in the research of Seo et al. [56] and Yap et al. [58], in which the results of PXRD structural studies provided evidence of complex formation between RA and GalαCD or HPβCD, respectively. The diffraction patterns of the physical mixture of RA and GalαCD or HPβCD were found to correspond exactly to the simple sum of the pure substances’ diffractograms. On the other hand, the crystalline patterns of RA in the RA/GalαCD or RA/HPβCD inclusion complex disappeared, indicating the complexation between these substances and the amorphous character of the obtained complexes [56,58].

Nuclear Magnetic Resonance (NMR) spectroscopy is one of the most versatile techniques for elucidating the structure and host–guest interactions in CD inclusion complexes. Depending on the physical state of the system, different NMR methods can be employed to provide complementary information on complex formation. Solution-state NMR techniques offer insights into the molecular mobility, geometry, stoichiometry, and inclusion mode of guest molecules within the CDs’ cavities. In contrast, solid-state NMR (ssNMR) is especially valuable for studying the crystallinity of complexes, guest conformations in CDs’ cavities, and intermolecular interactions between host–guest molecules in the solid state. The 1D NMR techniques of solution-state NMR, such as ^1^H and ^13^C NMR, are commonly used as a first step in CD inclusion complex studies. The chemical shift values of signals and signal broadening upon complexation can indicate the inclusion process, changes in the local environment, and the dynamic behaviour of both host and guest [79]. For more detailed spatial characterization, 2D techniques, especially ROESY NMR (Rotating-Frame Overhauser Effect Spectroscopy), are utilized. The ^1^H-^1^H ROESY method reveals through-space dipolar interactions between the protons of the guest and of the inner side of the CD’s cavity (H3 and H5), providing direct evidence of inclusion and allowing the determination of the orientation and/or the conformation of the guest molecule in the CD’s cavity [79]. In order to study CD complexes in the solid state, the ^13^C CP/MAS (^13^C Cross-Polarization Magic Angle Spinning) NMR method can be applied. This method is highly informative for characterizing inclusion in the absence of molecular mobility, revealing changes in the carbon environments of both host and guest molecules. It is particularly useful in confirming complex formation and evaluating the degree of order or amorphousness in solid forms [80]. Together, solution- and solid-state NMR techniques provide a comprehensive understanding of CD inclusion complexes, both in dynamic solution systems and in structurally constrained solid states.

In the research conducted by Munoz-Botella et al., an attempt to use the ^1^H NMR technique to quantitatively determine the association constants and stoichiometry was made. As NMR techniques are quantitatively less sensitive than UV–Vis absorption, for the analysis of the complexes, both the CD and the VA derivative concentrations used by the authors were elevated relative to those in the UV-Vis experiments. The signals associated with the protons of the CDs’ cavities were influenced by the presence of guest molecules and variations in their concentration. Consequently, the distinct peaks associated with the H6a and H6b protons in the pure CD samples converge into a singular signal for all inclusion complexes, irrespective of the type of complexed retinoid. Nevertheless, chemical shift value changes in merely 0.02–0.04 ppm precluded the quantitative assessment of the association constants [48]. Following the above publications, many other researchers also concluded about the formation of complexes with CDs based on subtle changes in the chemical shifts of the 3H and 5H protons in ^1^H NMR spectra [49,52,57,62].

Some authors, like Weisse et al. [57], also performed 2D ^1^H-^1^H ROESY NMR experiments in order to identify the protons of the VA propionate molecule involved in the inclusion phenomena, specifically those exhibiting cross-peaks with the inner protons of the CD. The cross-peaks demonstrated a spatial proximity of these protons (<4Å). The 2D spectra exhibited pronounced cross-peaks between the aliphatic protons of VA propionate and the H3 and H5 protons of the CD. Consequently, it was assumed that the aliphatic cycle of VA propionate participated in the inclusion process, whilst the vinyl group seemed to remain external to the cavity [57].

Although solution NMR is the most common NMR technique used in the studies of CD complexes with VA derivatives, some authors applied ssNMR for detailed structure analysis, e.g., the formation of the BC/βCD and BC/OSβCD in the work of Niu et al. [67] was evidenced by ^13^C CP/MAS NMR. Authors found that βCD molecules had an asymmetric conformation in the crystalline state, as the strong splitting for all the carbon atom resonances from each glucopyranose molecule was observed. In contrast, for the BC/βCD and BC/OSβCD inclusion complexes, the resolved resonances disappeared. In addition, a minor resonance peak located at 29.56 ppm, attributed to the resonance signal of C-16(16′) and C-17(17′) of the BC guest molecule, was observed in the spectra of the BC/βCD and BC/OSβCD inclusion complexes, in contrast to the physical mixture. Furthermore, the ^13^C signals of the BC/βCD and BC/OSβCD inclusion complexes produced sharp singlets compared to the physical mixture. In conclusion, the aforementioned results demonstrated that the βCD and OSβCD molecules within the inclusion complexes assumed a more symmetric conformation, with the carbon atoms of each glucopyranose unit situated in analogous environments, further suggesting that βCD and OSβCD established a unique channel-type structure for complexation with BC molecules [67].

Thermogravimetric Analysis (TGA) and Differential Scanning Calorimetry (DSC) are complementary thermal analysis techniques widely used to characterize the CD’s inclusion complexes, particularly with poorly soluble or thermolabile compounds, such as fat-soluble vitamins. While TGA monitors mass changes as a function of temperature, providing insights into thermal stability, composition, and water content, DSC measures heat flow, revealing thermal transitions, such as melting, crystallization, or complex formation [54,63,81]. TGA is particularly useful for evaluating guest content, estimating complex stoichiometry, and differentiating between physically adsorbed and molecularly encapsulated guest molecules. A shift in the decomposition temperature or the appearance of a distinct thermal degradation profile compared to the free guest often confirm successful inclusion. In turn, DSC provides evidence for interaction between the host and guest through the disappearance, shifting, or broadening of the melting endotherm of the guest. The absence of a sharp melting peak of the guest compound in the thermogram of the complex is often considered a strong indicator of encapsulation within the CD’s cavity. Additionally, DSC can help distinguish between amorphous and crystalline forms and detect the presence of residual uncomplexed material. Taken together, TGA and DSC offer valuable insights into the thermal behaviour, inclusion efficiency, and physical state of both the guest and the complex, aiding in the rational design of stable pharmaceutical or nutraceutical formulations.

In the research of Sapino et al. [44], the DSC method confirmed the formation of inclusion complexes between VA and EγCD or OγCD by the modification of the guest’s molecule endothermic peak. The endothermic signal around 50 °C in the physical mixture samples corresponded to VA melting point that did not appear in the thermograms of the complexes. These results indicated that intermolecular host–guest complexation occurred [44].

Villanova and collaborators used TGA measurements to assess the thermal stability of the encapsulated VA palmitate. The thermogram of the complexed VA ester was compared with those of the free molecule and the empty βCD. The curve of pure βCD indicated an initial minor loss (12%) at low temperatures, attributable to the loss of water molecules within the cavity. Subsequent to this loss, βCD demonstrated stability until approximately 280 °C, at which point the thermal degradation started. The free VA palmitate commenced degrading at approximately 112 °C. The degradation profile of the complex also showed a first loss step at low temperatures; however, it was less pronounced (8%) than that observed for the pure βCD sample, suggesting that certain water molecules were efficiently substituted by vitamins. In contrast, for the inclusion complex, a significantly reduced decomposition rate was noted. Approximately 20% of VA palmitate degraded within the initial hour of exposure. This degradation was mainly attributed to the decomposition of free VA palmitate molecules in solution. Subsequent to the initial hour, the concentration of VA palmitate remained quite stable for a minimum of 6 h. The enhancement in the stability of the encapsulated vitamin may be ascribed to steric hindrance; the isomerization of the double bond from trans to cis would be sterically impeded, thereby inhibiting the generation of additional decomposition products [47].

Computational methods are increasingly employed in the study of CD’s inclusion complexes to gain detailed insights into host–guest interactions, binding energetics, and structural conformations that are often challenging to fully resolve experimentally. Among the in silico methods used in CD complexes’ analysis are molecular docking, molecular dynamics (MD) simulations based on molecular mechanics (MM), quantum mechanics (QM)-based methods at various levels of theory (semiempirical, DFT, HF, MP) or mixed methods MM/QM (ONIOM and CP2K) [82,83]. Molecular docking is a fast and efficient technique to predict the most favourable binding pose of a guest molecule within the CD’s cavity. It provides preliminary insights into inclusion geometry, binding orientation, and affinity scores, which can be used for ranking potential complexes or comparing the binding preferences of similar molecules [84]. Molecular dynamics (MD) simulations go a step further by modelling the time-dependent behaviour of the host–guest system in an explicit solvent environment. MD offers information on complex stability, conformational flexibility, and solvent-mediated interactions over nanosecond to microsecond timescales. It is particularly valuable for assessing the dynamics of encapsulation, guest release mechanisms, and the persistence of key hydrogen bonds or van der Waals interactions [85]. Molecular mechanics (MM) approaches, typically using force fields such as AMBER or CHARMM, enable efficient energy minimization and the calculation of non-covalent interaction energies, helping to estimate binding free energies in a computationally accessible way. These methods are useful for optimizing docked poses or analyzing snapshots from MD trajectories [86,87]. Quantum mechanics (QM) methods, including density functional theory (DFT), offer the highest level of accuracy, enabling the detailed analysis of electronic interactions, charge distribution, and hydrogen bonding within the inclusion complex. Although computationally more demanding, QM calculations are essential for accurate energy decomposition, especially when studying small guest molecules or validating MM-derived models [88]. The integration of molecular docking, MD, MM, and QM enables a multi-scale computational approach that complements experimental techniques and supports the rational design of CD-based delivery systems [89].

Yap et al. [58] used molecular modelling with the MMFF94s force field (SYBYL) to predict the preferred orientation of 13-cis-RA in the CD cavity and identify the principal structural features that enhance its solubility and photostability. The energy scores derived from the computational study were determined to accurately represent the stability constants of the CD complexes acquired in the phase-solubility studies. Molecular modelling was conducted to elucidate the complexation modes of the 13-cis-RA/αCD and 13-cis-RA/HPβCD complexes. The molecular modelling of the 13-cis-RA/HPβCD complex demonstrated that the side chain of 13-cis-RA was incorporated into the oligosaccharide ring of CD, hence reducing isomerization due to the steric barrier exerted by the CD cavity. These findings were consistent with the results of the photostability study [58].

In turn, Fathalla and co-authors employed molecular docking (Molecular Operating Environment) to visualize the RA:CD possible binding sites and estimate the binding constants. The results showed that stable complexes with energies of −5.9 and −5.0 kcal/mol were theoretically formed upon solubilizing RA with both HPβCD and βCD, respectively. Single H-bond formation was detected with both CDs, enhancing the stability of the obtained complexes. Nevertheless, a reduced bond length and, consequently, enhanced stability were reported for the complex with HPβCD (1.77 Å, energy of −5.7 kcal/mol), and offered a potential explanation for the improved solubility observed empirically with HPβCD. Nevertheless, the lack of hydroxypropyl groups in βCD could impede the hydrophilic group from penetrating deeper into the CD cavity to adjust the carboxylic tail in the proper spot for such H-bonding construction, thereby failing to position the carboxylic tail appropriately for optimal hydrogen bonding. This also led to a marginal reduction in the bond strength (bond length of 2.47 Å) and diminished the stability of the resultant complex (energy of −5.04 kcal/mol), which elucidated the inferior solubility observed experimentally with βCD [54].

In the research by Ascenso and co-workers, molecular modelling calculations (DFT, B3LYP, 3–21 G basis set, Gaussian) were employed to ascertain the most favourable orientation of RA within the DMβCD cavity, resulting in a complex structure with the highest stability. The obtained data suggested that the structure of RA incorporated into the CD torus corresponded to the side chain containing the functional group COOH. The energy of this stable complex was −5723.432 hartrees, with a complexation energy of −32.5 kcal/mol, and it was lower than that of the complex where the RA ring was situated within the CD cavity (−5723.399 hartrees and −11.9 kcal/mol, respectively). The improved complex stability resulted from the hydrogen bonds established between the COOH group of RA and the protons at the bottom of the CD cavity (distance of 1.6 Å). Furthermore, the authors determined that the incorporation of the alkyl chain within the CD cavity enhanced photostability by reducing the likelihood of isomerization. The calculation results indicated that the complex of RA with two DMβCD molecules (1:2 stoichiometry) was energetically unfavourable, exhibiting a positive complexation energy of approximately 100 kcal/mol, which was further confirmed by experimental studies (DSC, PXRD, FTIR, AMF, Raman spectroscopy, and ^1^H NMR) [69].

Dynamic light scattering (DLS), also known as Photon Correlation Spectroscopy (PCS), is a non-invasive technique used to measure the hydrodynamic diameter and size distribution of particles or molecular assemblies in solution based on fluctuations in light scattering caused by Brownian motion. In studies of CD inclusion complexes, DLS is used to monitor the particle size changes upon complexation, the formation of nanoaggregates, and the colloidal stability of the resulting systems. When a guest molecule is encapsulated within a CD cavity, the overall size and solvation properties of the complex may differ significantly from the free guest or CD alone. DLS can detect these differences by measuring changes in particle size or the appearance of new populations, often indicating complex formation, self-assembly, or nanoaggregate formation in the case of hydrophobic guests [90]. Additionally, DLS can also track guest encapsulation efficiency and aggregation behaviour over time or under varying pH and ionic strength conditions. Although this method does not provide direct structural information, it is a valuable complement to spectroscopic and calorimetric methods, especially when studying inclusion complexes intended for biomedical or pharmaceutical applications in the nanoscale range [91].

In the studies of VA derivatives, the DLS technique was used in the research conducted by Ascenso et al. [51] in order to compare the differences between the mean diameter and the size distribution of RA/DMβCD ultradeformable vesicles and RA ultradeformable vesicles without DMβCD for further skin penetration (UDV = phospholipid vesicles with lipid bilayers). The final mean size distribution was approximately 150 ± 50 nm for both formulations, as expected. In addition, the unimodal mean diameter distribution (polydispersity index, PI) met the quality criteria (it was below 0.2) [51]. In the studies of Weisse’s research group, DLS was applied to assess the size distribution and the stability of the nanocapsules obtained using a new cholesteryl-cyclodextrin derivative and highly unstable and poorly water-soluble VA propionate. Due to the stabilizing effect of the surfactant, the authors were able to form 130 nm diameter nanocapsules with a low degree of polydispersity and spherical shape. Formulations showed rapid sedimentation (inferior to 4 days) and the size increased due to the aggregation of the particles [74].

#### 3.1.3. Applications/Aim of Obtaining VA Derivative Inclusion Complexes with CDs

The main reason for obtaining the inclusion complexes of VA derivatives and CDs is to increase the solubility of these oily, highly hydrophobic substances [39,44,47,52,53,54,56,57,58,60,66,71,72,75]. Another aim for complexation with CDs is to improve the stability of this vitamin and enhance its resistance to decomposition under the influence of light, oxygen, and temperature [39,44,47,49,53,58,59,61,62,63,65,67,68,72]. Some studies are concerned with the development of a new formulation, consisting of CD complexes with VA analogues with better bioavailability, loading capacity, prolonged shelf life, and antioxidant properties [44,45,51,59,60,74,75]. All publications on VA derivative complexation with CDs are summarized in Table S1 (in Supplementary Materials), and the most interesting applications of VA derivative inclusion complexes with CDs are presented below in more detail.

**Figure 5:**
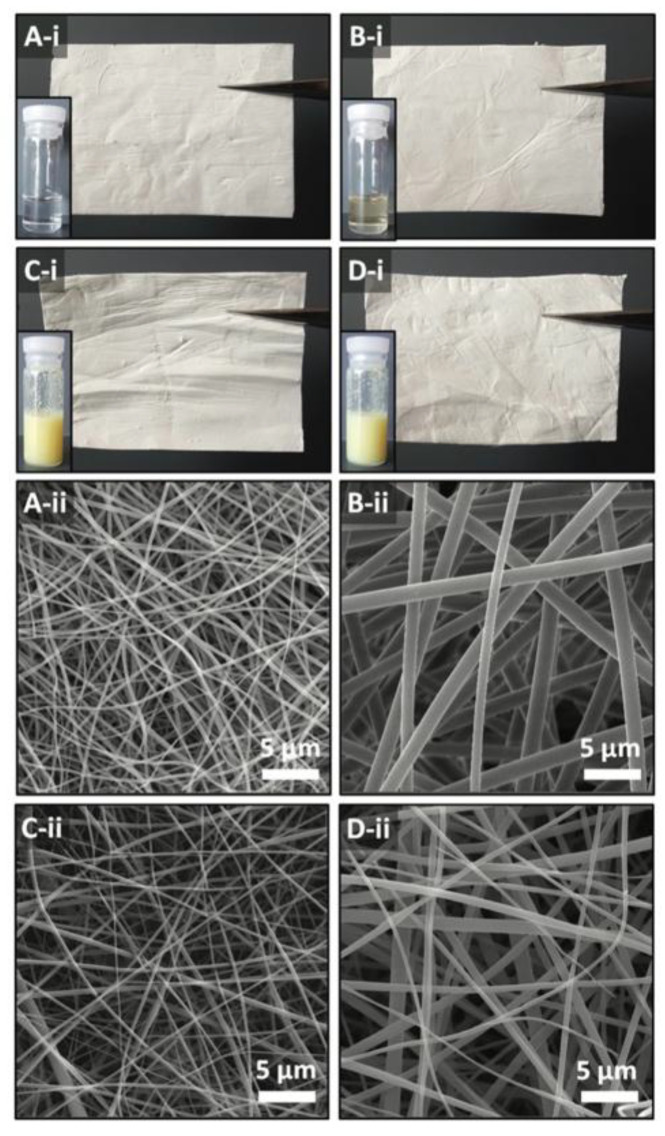
(**i**) Pictures of the electrospinning solutions and the self-supporting webs, and (**ii**) representative SEM pictures of (**A**) HPβCD NW, (**B**) HPγC NW, (**C**) VA acetate/HPβCD NW, and (**D**) VA acetate/HPγCD NW. Reproduced/adapted from [45] with permission from The Royal Society of Chemistry.

VA acetate, one of the commonly known and used derivatives of VA, has antioxidant properties, but due to its lipophilic properties, it has very limited solubility in water and is very sensitive to oxidation and high temperature. The encapsulation of VA acetate into CD inclusion complexes’ nanofibers is one of the methods to bypass this problem and an example of the development of the innovative carriers and delivery systems of fat-soluble vitamins with rapid oral dissolution capabilities. The nanofibers of VA acetate inclusion complexes with the hydroxypropylated derivatives of βCD, namely HPβCD and HPγCD, were synthesized from polymer-free aqueous environments via the electrospinning process. The final vitamin acetate/CD nanofiber webs were produced with a VA acetate loading capacity of 5% (*w*/*w*). The amorphous distribution of VA acetate within the nanofibrous webs, achieved through inclusion complexation, along with the distinctive characteristics of nanofibers (such as a high surface area and porosity), facilitated the rapid disintegration and dissolution/release of VA acetate in a simulated saliva and aqueous environment. The increased solubility of VA acetate in the VA acetate/CD NW formulation also resulted in higher antioxidant properties of this molecule, when compared to “free” VA acetate. Furthermore, in the newly designed form, VA acetate exhibited thermal degradation at elevated temperatures when compared to the noncomplexed one, indicating the improved thermal stability of this active molecule. In the described work, HPβCD formed inclusion complexes more favorably than HPγCD. Consequently, noncomplexed VA acetate crystals were observed in the VA acetate/HPγCD NW, whereas VA acetate molecules included in the VA acetate/HPβCD NW were entirely in complexed and amorphous forms (Figure 5). Consequently, improved solubilization efficacy, increased release quantity, and augmented antioxidant capabilities were observed for the VA acetate in the form of the VA acetate/HPβCD NW [45].

**Figure 6:**
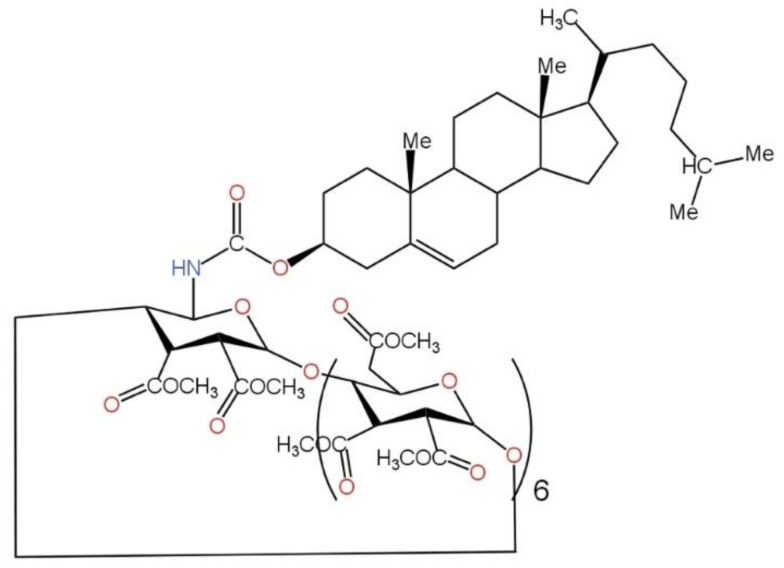
Chemical structure of Heptakis(2,3-di-O-acetyl)-hexakis-(6-o-acetyl)-6I-(cholest-5-en-3-yloxycarbonyl) amino-6I-deoxycyclo-maltoheptaose (Chol-βCD-Ac).

VA derivatives play a crucial role in epithelial proliferation, making them of significant interest to the pharmaceutical and cosmetic sectors. Nonetheless, their molecules are insoluble in water and highly unstable when exposed to light and oxygen, which restricts their application. Therefore, creating stable forms, resistant to the destructive effects of external factors, is extremely important. Improving the stability of one of the VA derivatives (VA propionate) via nanocapsulation with a novel cholesteryl-acetyl-β-cyclodextrin derivative (Chol-βCD-Ac, Figure 6) was the subject of Weisse et al.’s [74] research. The lipidic character of VA propionate facilitated the creation of nanocapsules with a consistent size distribution and prolonged stability, as confirmed by dynamic light scattering (DLS) analysis. The obtained colloidal suspension can be utilized to create a gel that facilitates the entry of encapsulated VA propionate into the skin [74].

**Figure 7:**
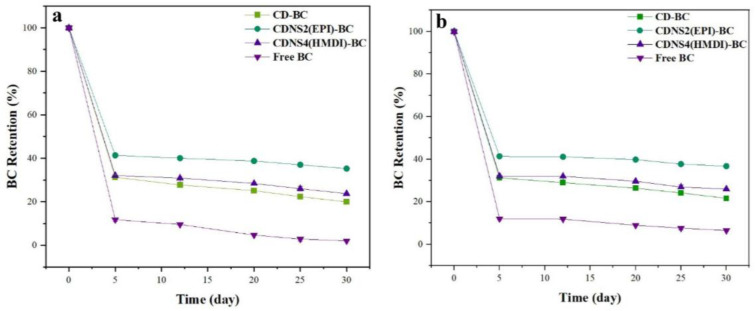
Storage stability of free and encapsulated BC within 30 days at 25 °C under light (**a**) and in the dark (**b**). Reproduced from [75] with permission from SNCSC.

β-carotene (BC) is a precursor of VA in the human organism and possesses potential anticancer properties; however, its distribution is impeded by low solubility and storage instability. To address these problems, the study conducted by Yazdani et al. [75] examined the application of synthesized cyclodextrin-based nanosponges (CDNSs), utilizing varying proportions of two cross-linkers, epiclon (EPI) and hexamethylene diisocyanate (HMDI), to create an inclusion complex with BC. The optimized ratios of crosslinkers to βCD for the two most effectively encapsulated βCDNSs-BC were established as 2:1 for EPI and 4:1 for HMDI, yielding loading efficiencies of 61.46% and 59.61%, respectively. The characterization physicochemical experiments were meticulously conducted for the selected βCDNSs using UV-Vis spectroscopy, FTIR, DSC, PXRD, SEM, and DLS. The two most effective complexes were also evaluated for drug solubility, in vitro release, storage stability, photostability, scavenging capacity, and in vitro cytotoxicity. Encapsulation enhanced the solubility by about 10-fold and increased 30-day storage stability by 40% relative to native BCs. The βCDNS with the EPI cross-linker enhanced the drug solubility more significantly than CDNS(HMDI) and more effectively inhibited drug degradation during a 30-day storage period (Figure 7). On the other hand, the CDNS(HMDI) has a superior in vitro release at physiological pH settings. The free-radical scavenging capability has been improved in CDNSs-BC relative to BC. The MTT assay demonstrated a differential enhancement in cytotoxicity in both normal and cancer cells when treated with CDNSs-BC in comparison to free BC. CDNS4(HMDI)-BC exhibited higher cytotoxicity against cancer cells than CDNS2(EPI)-BC. The formation of the inclusion complexes of CDNSs with BC has demonstrated potential for enhancing the stability and solubility of BC. This delivery technique could markedly enhance the insufficient bioavailability of BC, leading to reduced dosage requirements. The authors concluded that their study illustrates that βCD nanosponges cross-linked with HMDI and EPI serve as two promising carriers for the delivery of BC [75].

### 3.2. Complexes of Vitamin D and Its Derivatives with Cyclodextrins

#### 3.2.1. Preparation Methods of Inclusion Complexes with VD Derivative Complexes with Cyclodextrins

The complexes of VD and its analogues with cyclodextrins were usually prepared in a molar ratio of 1:1 or 1:2 [42,55,73,92,93,94,95,96,97,98,99]. However, cases of a larger number of cyclodextrin molecules per one molecule of VD derivative can be found, i.e., 1:2,5 [20], 1:4 [100], 1:5, and 1:10 [20]. The main methods for obtaining the complexes of VD and its derivatives with CDs include the following: kneading with a small amount of solvent (water or ethanol) [55,96], milling [100], freeze-drying [94], spray-drying [96,98], co-precipitation [42,73], the evaporation of ethanolic–aqueous solution [20,96,101,102], microwave radiation [93], and complexation in ethanolic–aqueous or aqueous solution [95,96,103]. The VD analogues most often formed complexes with βCD [20,42,55,93,95,96,101,102,103] or its modified derivatives, such as MβCD [97], HPβCD [20,94,98], and GluβCD [73]. Less frequently, other native CDs, such as αCD [20,97] and γCD [20,103], were used.

Most often, CDs were used for complexation as pure substances; however, some researchers created complexes of VD derivatives with, e.g., βCD-nanoliposomes (βCDNLPs) [95], or βCD-based nanosponges [100]. Several authors applied βCD and its analogues, such as DMβCD and HEβCD, as mobile-phase modifiers in the study on improving the chromatographic separation of VD derivatives [104,105,106,107]. Most often, the binary complexes of VD and CDs were investigated, but there are also a few cases of ternary complexes of VD3/βCD with metal ions such as Al^3+^, Co^2+^, Cu^2+^, and Zn^2+^ in studies on the development of novel controlled release systems [101,102].

#### 3.2.2. Methods of Analysis of Inclusion Complexes with VD Analogues

In order to analyze the structures and properties of the obtained inclusion complexes of VD and its derivatives and CDs, various methods were used. The most commonly used analytical techniques in the study of VD/CD complexes can be divided into spectroscopic methods, such as FTIR, NMR, UV-Vis, PXRD; thermal analysis techniques, such as DSC, TGA; microscopic methods for surface morphology studies, such as SEM; and computational methods, such as molecular mechanics, and based on this—molecular dynamics. General information about these methods, and about the features of the structures and properties of CD inclusion complexes that can be obtained by them, is described in detail in Section 3.2.1. This subsection will focus only on the specific examples of the application of these in the study of VD/CD complexes described in scientific papers.

Many studies applied the FTIR method in order to confirm the formation of VD/CD complexes by comparing their vibrational spectrum with the spectra of pure substrates and their physical mixtures prepared in proper molar ratios. Usually, the spectra of physical mixtures are the superposition of the spectra of pure substrates, while the FTIR spectra of complexes differ slightly in the number of vibrations in the spectrum and their position (differences in wavenumber values). Although the registration of FTIR spectra does not require complicated sample preparation and is not difficult to perform, the obtained results are usually not very informative, and the formation of complexes should often be confirmed by additional methods, e.g., NMR, PXRD, or DSC [20,93,94,98].

More information on the formation of complexes and their structure can be provided by solution NMR [93,96,102,104]. In research conducted by Bakirova et al. [93], ^1^H and ^13^C NMR spectra confirmed the formation of VD3/βCD complexes. The resonances of pure and complexed βCD showed a pronounced chemical shift towards a strong field. The largest differences in the *δ* values in the VD3/βCD ^1^H NMR spectrum were characteristic for the H3 and H5 protons that are located inside the CD cavity. In the case of the ^13^C spectrum, there was also a difference in the chemical shift values (from 0.06 to 0.22 ppm). The proportional increase in the chemical shift in the ^1^H and ^13^C NMR spectra was observed with an increase in the VD3 concentration due to the shift in the equilibrium state towards the formation of an inclusion complex and showed that hydrophobic interactions were the driving forces for the inclusion complex formation [93]. UV-Vis spectroscopy is an essential technique for studying inclusion complexes between VD derivatives and CDs. By analyzing changes in the UV spectra upon complex formation, it provides valuable information on the interaction between the host and guest molecules. This technique was applied to determine the encapsulation efficiency and solubility improvement due to complexation, aiding in the characterization of the complexes of CDs with VD analogues [20,94,101].

The PXRD method was frequently employed to confirm VD/CD complex formation by comparing the diffraction patterns of pure components and complexes. Moreover, the discussed method was also useful in detecting changes in the crystallinity [20,42,96,98]. An example of the application of PXRD in studies of VD/CD complexes was the research carried out by Wang and co-authors. The sharp peaks at 2θ of 5.1°, 6.7°, 13.7°, 15.7°, 18.1°, and 21.8° in the spectrum of pure VD3 illustrated the crystalline nature of the compound. The diffraction pattern of the host HPβCD molecule demonstrated a broad halo in the 15–25° (2θ) range, indicating the amorphous character of the sample. The PXRD pattern of the VD3 and HPβCD physical mixture appeared as the superposition of VD3 and HPβCD X-ray patterns. The sharp peaks at 15.7°, 18.1°, and 21.8° revealed the VD3 crystalline structure in the mixture. For the VD3/HPβCD complex, the PXRD pattern showed a large and broad peak similar to the HPβCD sample, indicating the amorphous character of the spray-dried material [98].

The DSC method applied in the studies of VD/CD complexes measured heat flow, revealing thermal transitions such as melting, crystallization, or complex formation [20,93,94,97], whereas TGA monitored mass changes as a function of temperature, providing insights into thermal stability, composition, and water content [93]. The application of the DSC method in CD complexes’ analysis is useful due to the phenomenon that when a guest molecule is included in a CD cavity, its melting point, boiling point, and sublimation point shift to a different temperature or may disappear entirely and therefore surely confirm the complex formation [58]. Due to the fact that VD3 has a low melting point (84.5 °C), it is susceptible to structural instability and degradation as the temperature increases. Carrier molecules that can bind VD3 and improve its thermal resistance are therefore beneficial options. Braitweite et al. [20] observed extensive differences between the DSC thermograms of VD3/CD inclusion complexes and pure substrates used in their formation. The VD3/αCD thermogram exhibited no discernible endothermic peaks or melting points. The change in the peak size from a large peak at 135 °C on αCD’s DSC thermogram to a flattened one on the thermogram of the VD3/αCD sample indicated the formation of a complex in which VD3 substituted water molecules inside the αCD cavity. Furthermore, the distinctive melting point of VD3 was absent in the thermogram of VD3/αCD. The greater area under the curve for the inclusion complex suggested that more thermal energy was required to induce a phase change, suggesting the enhanced stability of the complex sample. Two glass transition temperatures (Tg) were observed in both the physical mixture and αCD, which were absent in the profile of the VD3/αCD inclusion complex. The shallower and broader endotherms observed for the VD3/βCD complexes, in comparison to pure βCD, corresponded to reduced water loss. The observed discrepancies suggested distinct solid-state structures, further corroborated by a crystallization or oxidation exotherm at around 220 °C for pure VD3, which was absent in VD3/βCD. The absence of the VD3 oxidation peak in the VD3/βCD complex indicated that the vitamin was shielded from oxidation when associated with βCD [20].

The SEM technique was utilized to investigate the particle size and morphology of the VD/CD complexes [42,93,98]. Bakirova and co-workers studied the formation of the VD3/βCD inclusion complexes with a molar ratio of 1:2 using the SEM method. The comparison of sample surfaces showed significant differences between pure βCD, the physical mixture of VD3 and βCD, and the inclusion complex VD3/βCD. The SEM pictures revealed that βCD exhibited a crystalline rhombic structure, while VD3 displayed irregular-shaped particles. In the physical mixture, the characteristic particles of VD3 and the parallelogram structure of βCD existed coincidentally and separately, showing no change in the physical forms of the individual substances. In contrast, the inclusion complex presented a remarkable change in the morphology and the shape of the separate particles of VD3, and βCD was no longer indistinguishable. Modification in the shape of particles suggested the formation of the VD3 inclusion complex with βCD [93]. Similar results were obtained by Wang et al. [98] in studies on the VD3 complex with HPβCD. SEM images revealed the differences in the microstructures of pure VD3, HPβCD, their physical mixture, and a spray-dried VD3/HPβCD complex. The crystal structure of VD3 had a needle shape, while HPβCD’s image was typical for amorphous materials. In the SEM photo of the physical mixture, the crystalline VD3 could be found intermixed with HPβCD. However, the inclusion complex VD3/HPβCD displayed the typical morphology of spray-drying amorphous materials, and the particle size was less than 10 µm. The comparison of these four images showed that the complex had a different microstructure compared toVD3, HPβCD, and their physical mixture, which confirmed the formation of the inclusion complex and its amorphous nature [98].

Molecular modelling is a powerful approach for studying the inclusion complexes between VD derivatives and CDs. Techniques such as molecular mechanics and molecular dynamics simulations allow for the visualization and analysis of host–guest interactions at the atomic level. These methods provide insights into the structural organization, binding affinity, and dynamic behaviour of the complexes, offering a complementary perspective to experimental techniques and aiding in the optimization of VD delivery systems.

In the research by Braithwaite et al. [20], molecular mechanics (MM+) simulations for VD3/CD inclusion complexes were executed to identify the most energetically stable complex, elucidate the binding/inclusion mode of VD3 with the corresponding cyclodextrin (CD), and create a space-filling model that illustrated the optimal geometric conformation. VD3 was introduced vertically into the cavity of each CD via its aliphatic and aromatic ends (inserted from the wider end of the CD). The resulting structures were then energetically minimized using the AMBER3 force field. MM+ simulations allowed for the analytic–mathematical representation of the potential energy surfaces and provided data related to the contributions of bonding (bond stretching, bond angle, and torsional contributions), arising from deviations from optimum dihedral angles and non-bonding energies such as van der Waals interactions, H-bond energy functions, and electrostatic energies towards the geometrical stabilization of the VD3/CDs complex. Despite the optimized VD3/αCD structure achieving a final total steric energy stabilization of 22 kcal/mol, all bonding interactions destabilized the VD3/αCD structure due to steric hindrances arising from the small molecular cavity of αCD, which could not accommodate the steric volume of VD3. Conversely, VD3/γCD experienced partial stabilization from both bonding and non-bonding attributes. VD3/γCD, however, was destabilized by torsional contributions and electrostatic interactions. The varied behaviour and elevated stabilization energy (24 kcal/mol) resulted from the strain-free oscillation of the VD3 molecule within the CD cavity, facilitated by its larger volume, allowing the molecules to orientate with respect to the surrounding molecular groups; VD3/βCD was most stabilized among the unmodified CD complexes (α-, β-, and γCD). However, almost all energy stabilization was attributed to the non-bonding steric component (van der Waals forces), hence emphasizing the need for an optimal fit of the molecule inside the CD cavity. The total ΔE for VD3/βCD exceeded that of VD3/αCD and VD3/γCD, which possess lower and higher cavity volumes, respectively; this proved the presence of a region-of-maximum in terms of the spatial fit among the individual molecules. The novel in silico molecular simulations elucidated the experimental and physicochemical characterization necessary for the effective creation of VD3 inclusion complexes with various CDs [20].

Ferro-Costas et al. [99] conducted research to elucidate the catalytic mechanism of βCD in the formation of VD from preVD3, providing a computational analysis of experimental findings that demonstrated a 40-fold acceleration in the isomerization reaction of PreVD3 to VD3 when it occurred in a βCD’s dimer compared to the reaction in conventional isotropic solutions. This work used molecular dynamics (MD) simulations and the statistical theory of multistructural transition states to elucidate the basis of the observed acceleration. It was demonstrated that the conformational landscape in the isomerization of PreVD3 was strongly dependent on whether the system was closed. In isotropic environments, the triene moiety of PreVD3 had significant torsional flexibility. However, following confinement, such flexibility was limited to a more constrained conformational area in complex with βCD. In both scenarios, the computed rate constants closely aligned with the experimental findings, enabling one to ascertain that the flexibility limitation of PreVD3 was the primary catalytic component. The findings presented by the authors of this theoretical study enhanced the understanding of VD3 isomerization and underscored the significance of molecular dynamics in biochemical modelling [99].

#### 3.2.3. Applications/Aim of Obtaining VD Derivative Inclusion Complexes with CDs

The main goal for preparing the inclusion complexes of VD derivatives and CDs is to improve the aqueous solubility, which in turn leads to increased bioavailability and therapeutic efficacy [20,73,93,94]. Another reason for complexation with CDs is to enhance the resistance of VD analogues to decomposition under the influence of light, oxygen, and temperature [42,73,93,100]. Some studies concern the development of new formulations consisting of CD complexes with VD analogues with a better loading capacity or modified release [95,101,102], whereas others deal with the application of CDs for the better separation of VD analogues or metabolites [104,105,106,108]. An interesting direction of research on VD complexes with CDs is the study of the influence of the presence of CDs on the isomerization processes [97,99] or the acceleration of VD conversion to active metabolites by microorganisms [104]. All publications on VD derivative complexation with CDs are summarized in Table S2 (in Supplementary Materials), and the most interesting applications of VD derivative inclusion complexes with CDs are presented below in more detail.

CDs can serve as environmental catalysts, increasing the rate of reaction by forming inclusion complexes with guest molecules, e.g., lipid-soluble vitamins. Their action is based on changing the microenvironment, stabilizing the transition state, limiting movement of the molecule, and specific chemical interactions [109,110,111]. This ability of CDs was used to investigate the kinetics of the preVD-to-VD isomerization process in an aqueous solution of βCD by Tian et al. [97]. Their findings indicated that at 5 °C, the forward (k_1_) and reverse (k_2_) rate constants for the isomerization of preVD3 to VD3 were augmented by over 40 and 600 times, respectively, in comparison to those in n-hexane (k_1_: 8.65 × 10^−6^ versus 1.76 × 10^−7^ s^−1^; k_2_: 8.48 × 10^−6^ versus 1.40 × 10^−8^ s^−1^), representing the most rapid rate of this isomerization documented at this temperature. Thermodynamic investigations demonstrated that the equilibrium constant of the reaction diminished by almost 12-fold relative to that in n-hexane at 5 °C, while the percentage of VD3 at equilibrium escalated with a rising temperature in βCD. When complexed with βCD, the isomerization of preVD3 to VD3 became endothermic (ΔH = 13.05 kJ mol^−1^), in contrast to its exothermic nature in other media. The authors asserted that the thermodynamically unfavourable conformers of preVD3 are stabilized by βCD, hence enhancing the rate of isomerization. This conformation-controlled mechanism may significantly influence the regulation of the preVD3 to VD3 endocrine system in vivo, as observed in the sea urchin [97].

In numerous instances, the inclusion complexes of VD3 neglect the consideration of a third component, such as a metal ion [112]. However, Crespo-Biel et al. [113] delineated a supramolecular complex including βCD and a metal ion coordinated with ethylenediamine, whereas Torri et al. [114] elucidated the inclusion complex of progesterone and a metallic ion. The aim of the research by Mercê et al. [101] was to investigate whether the addition of a metal ion to the inclusion complex of βCD with VD3 would affect the stability and whether it would change the complexing properties of CD. The reagent quantities were selected to ensure a sufficient amount of βCD for the encapsulation of VD3. The authors tested several βCD: VD3 ratios, including 3:1, 2:1, and 1:1, without an observed result indicating complex formation. The supramolecular structure was observed to form only when the ratio reached a minimum of 5:1. Ultimately, all tests utilized 5:1 or 10:1 ratios of βCD to VD for binary systems. Thus, for ternary assemblies, the ratios of βCD: VD3: metal ion were 5:1:1 or 10:1:1. The researchers successfully created novel ternary assemblies, including βCD/VD3, and three metal ions, Co(II), Cu(II), and Zn(II). Through the application of ^13^C NMR, UV-Vis diffuse reflectance spectroscopy, and PXRD patterns, it was demonstrated that VD3 formed ternary complexes with βCD and all three studied metal ions. However, the ternary assemblages were detectable only under experimental conditions with a βCD: VD3: metal ion ratio of 10:1:1 or 5:1:1 at both a pH of 3 and 7. Therefore, it seems that the design of the synthesis of CD complexes with fat-soluble vitamins and divalent metal ions should be further investigated [101].

Another study described the synthesis of novel supramolecular assemblies of βCD, VD3, and Al (III) cations, utilizing previously established βCD: VD: metal ions ratios of 10:1:1 and 5:1:1. The study focused on solid complexes derived from aqueous or aqueous ethanol solutions, analyzed via ^13^C solid-state NMR and PXRD methods. The PXRD spectra of ternary assemblies indicated that they were channel-type inclusion complexes. The solution systems were analyzed by potentiometric titrations, leading to the calculation of binding constants and the determination of speciation based on pH variations [102].

**Figure 8:**
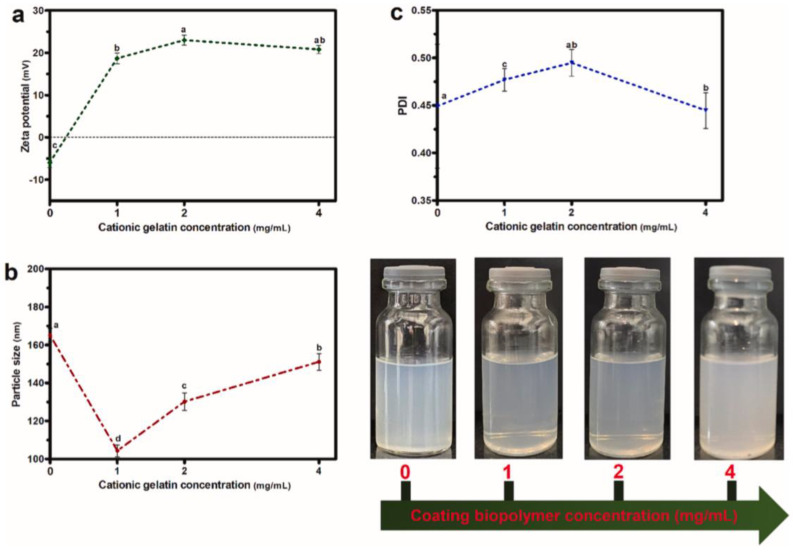
The changes in zeta potential (**a**), particle size (**b**), and PDI values (**c**) of NLPs by coating with different concentrations of gelatin. Data are expressed as mean ± standard deviation (n = 3), and the means followed by different lowercase letters are significantly different at the 5% level in Duncan’s test (*p* < 0.05). PDI: polydispersity index. Reprinted with permission from [95].

**Figure 9:**
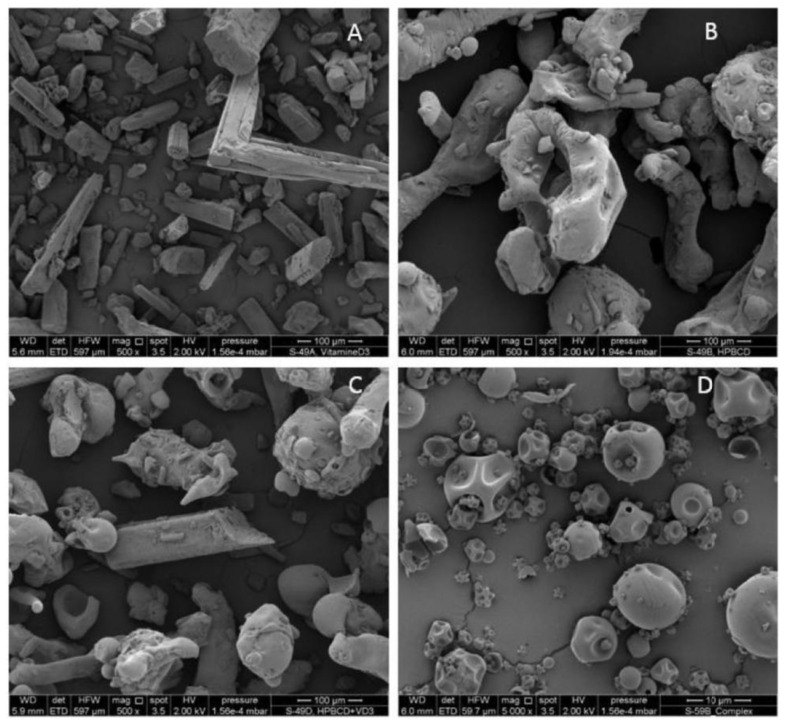
Field-emission scanning electron microscopy (FE-SEM) images of blank NLPs (**A**), the coated blank NLPs with 2 mg/mL of gelatin (**B**), the complex-loaded NLPs (**C**), and the coated complex-loaded NLPs with 2 mg/mL of gelatin (**D**). Reprinted with permission from [95].

**Figure 10:**
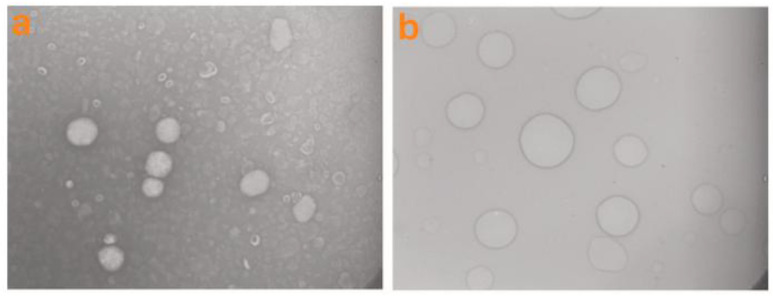
The transmission electron microscopy (TEM) images of the complex-loaded NLPs (**a**) and the coated complex-loaded NLPs with 2 mg/mL of gelatin (**b**). Reprinted with permission from [95].

The objective of the other work conducted on the topic of CD complexes with VD was to construct a βCD/VD3 inclusion complex and encapsulate it within gelatin-coated nanoliposomes (NLPs). The FTIR confirmed the formation of the βCD/VD3 inclusion complex. Subsequently, various gelatin concentrations (1, 2, and 4 mg/mL) were employed to surface-coat the blank NLPs. An appropriate gelatin concentration of 2 mg/mL was selected for coating the complex-loaded NLPs based on particle size, shape, and zeta potential considerations. The particle size and zeta potential of the coated complex-loaded NLPs were 117 ± 2.55 nm and 19.8 ± 1.25 mV, respectively (Figure 8). TEM and SEM pictures validated the development of a gelatin biopolymer layer surrounding the NLPs’ vesicles (Figure 9 and Figure 10). The encapsulation efficiency within the NLPs was 81.09%. The βCD/VD3 complex loaded NLPs and their coated variant demonstrated a regulated release profile under simulated gastrointestinal conditions. The authors concluded that the proposed methodology could be an innovative strategy for producing highly stable nanocarriers and that their findings could pave the way for stable nanoformulations aimed at enhancing the nutrition of individuals with VD3 insufficiency [95].

The competition between four bile acids (cholic, taurocholic, chenodeoxycholic, and lithocholic) and the fat-soluble vitamins VD_3_ and VA in the formation of βCD inclusion complexes was studied by Comini et al. [55]. The intermolecular interactions were observed using ^1^H NMR spectroscopy. Lithocholic and chenodeoxycholic acids exhibited a stronger affinity for βCD than cholic and taurocholic acids. The affinity of bile acids for βCD increased in relation to their hydrophobicity. It was found that the affinity of VA and VD_3_ for βCD was lower than that of the bile acids. Therefore, when lithocholic or chenodeoxycholic acids were present, the formation of βCD inclusion complexes with the vitamins did not occur. The results of this study suggested that the depletion of lipophilic vitamins would not occur upon ingestion of βCD, thus providing further support for the safety and suitability of βCD as an ingredient in foods and orally administered drugs [55].

Nanosponges are innovative, nanostructured materials with high porosity that can bind, store, and then release various substances, such as drugs, fragrances, or toxins, in a controlled manner. In the case of hydrophobic substances, such as fat-soluble vitamins, nanosponges facilitate their solubilization [115]. The improvement of VD bioavailability through complexation in a cyclodextrin-based nanosponge (CD-NS) was the subject of studies led by Uberti et al. [100]. VD is crucial for several cellular activities because of its capacity to attach to the vitamin D receptor (VDR), which is found in multiple tissues. Nevertheless, VD3 exhibits low bioavailability, prompting the exploration of several ways to enhance its absorption. In order to overcome this problem, βCD-NS 1:4 was obtained using mechanochemical method, and the complex formation was verified using FTIR-ATR and TGA. TGA revealed an enhanced thermostability of the complexed form of VD3. Subsequently, in vitro tests were conducted to analyze the biological activity of VD3 complexed in nanosponges on intestinal cells and to evaluate its bioavailability. The VD3 complexes augmented cellular activity in the intestines and promoted VD3 bioavailability. This study conclusively established the capacity of βCD-NS complexes to enhance the biological efficacy of VD3 [100].

Cyclodextrins (CDs) as mobile-phase additives in liquid chromatography (HPLC) are used not only for the separation of chiral compounds but also in other cases where their specific properties can improve the resolution, selectivity, and sensitivity of the analysis [108,116]. Shimada and co-authors decided to utilize the mentioned properties of CDs in their studies on the retention behaviour of VD and related compounds. The retention characteristics of VD1-VD5 and proVD2-VD5 were analyzed utilizing RP HPLC, employing MβCD as a mobile-phase additive. Reversed-phase HPLC was found to be superior to normal-phase HPLC in the separation of these analogues. It was found that the addition of MβCD into the mobile phase effectively separates the pair of VD2 and VD3 or preVD2 and VD3 [104]. The separation of DHVD3 metabolites was also investigated utilizing the HPLC method with CD as the mobile-phase additive. The addition of γCD to the mobile phase, with acetonitrile as the organic modifier, effectively separated 24,25-DHVD3 and its 3-epimer. The separation of the 25-epimers of 25,26-DHVD3 was partially achieved utilizing a mobile phase consisting of methanol and DMβCD as the organic modifier and additive, respectively. The aforementioned separation techniques were utilized for the identification of metabolites in the plasma of rats fed 25-HVD3 [105].

VD also plays an important role in the functioning of the immune system and in maintaining the integrity of the intestinal barrier. Research suggests that VD deficiency may be associated with the occurrence of irritable bowel syndrome (IBS) and the severity of its symptoms [117,118]. VD3 is essential for numerous cellular activities via its receptor interactions. The biological efficacy of VD3 can fluctuate depending on its solubility and stability. Therefore, the aim of a subsequent study conducted by Uberti et al. [119] was to enhance its biological effects by complexing it within βCD nanosponges (VD3/βCDNS). Consequently, its activity was assessed on two distinct gut–brain axes: the healthy gut/degenerative brain axis and the inflammatory bowel syndrome gut/degenerative brain axis. VD3/βCDNS alleviated lipopolysaccharide-induced damage (100 ng/mL; during 48 h), reinstating the viability, integrity, and functionality of tight junctions while diminishing reactive oxygen species production, lipid peroxidation, and cytokine levels. Subsequent to intestinal transit, VD3/βCDNS improved the neurodegenerative state in both the healthy axis and the IBS model, indicating VD3/βCDNSs’ capacity to maintain efficacy and advantageous effects even under IBS settings. This study illustrated the efficacy of the novel VD3 form, VD3/βCDNS, in influencing the gut–brain axis under both healthy and compromised conditions, highlighting a 50% augmentation in the biological activity of VD3 due to complexation, which amplified its beneficial effects in the gut and brain [119].

Cyclodextrin inclusion complexes incorporating protein hydrolysate peptides and fat-soluble vitamins are utilized for this purpose. Alongside the synthesis of βCD nanocomplexes with fat-soluble VD3 and VA, the research conducted by Kurchenko and co-workers [120] sought to establish a method for producing hypoallergenic peptides from the enzymatic hydrolysate of whey proteins and their incorporation into βCD. The synthesized clathrates were subsequently employed to build multicomponent compositions for functional nutrition. The analytical techniques employed in order to study multicomponent compositions were fluorimetry, electrophoresis, TGA, and HPLC-MS. Inclusion complexes with fat-soluble vitamins, i.e., VD3/βCD and VA/βCD, were successfully converted from an olive oil solution to a water-soluble powder form. The toxicological investigations on *Tetrahymena pyriformis* found that the toxicological and hygienic evaluation of the nanocomplexes, VD3/βCD and VA/βCD, along with their multicomponent composite, classified them under hazard class 5 (non-hazardous chemicals) based on the average lethal dose. In summary, fat-soluble vitamins, along with peptides generated in powder form, can be easily dosed and employed to formulate various functional food products (e.g., for infants). Complexation with CDs converts fat-soluble VD3 and VA into a powdered form suitable for use in multicomponent formulations.

Among the innovative approaches to fortifying food with VD, fermentation is a new strategy. Some microorganisms, such as *Amycolata* sp., *Rhodococcus erythropolis,* can convert VD3 into 1α,25-DHVD3 via 25-HVD3 during fermentation processes [121]. In a study performed by Takeda et al., [107] it was demonstrated that partially methylated βCD and γCD could augment the microbial hydroxylation of VD3 by *Amycolata autotrophica*, a bacterium from the genus of *Pseudonocardia*. The addition of partially methylated βCD (PMβCD) enhanced the productivity of 25-HVD3 approximately seven-fold relative to the absence of CD. The simultaneous application of PMβCD and γCD augmented the generation of 1α,25-DHVD3 in a tank fermenter by approximately sixteen times relative to the absence of CDs [107].

### 3.3. Vitamin E and Its Derivative Complexes with Cyclodextrins

#### 3.3.1. Preparation Methods of Inclusion Complexes with VE Derivatives

The complexes of VE and its derivatives with CDs were usually prepared in a molar ratio of 1:1 [122,123,124,125,126,127]. However, cases of a larger number of cyclodextrin molecules per one molecule of VE derivative can be found, i.e., 1:2 [123,126,127,128,129], 1:3 [73,126,129], 1:4 [130,131], 1:5 [20,132], 1:10 [20,132], or even 1:12, 1:16, 1:20, 1:25, and 1:30 [132]. Sometimes an excess of VE is used in relation to CDs, e.g., 1,5:1, 2:1, 2,5:1, 3:1, and 10:1 in the cases of inclusion complexes with large-ring CDs [125,129,133].

The main methods for obtaining the complexes of VE and its derivatives with CDs include the following: kneading with a small amount of solvent (water or water–methanol mixture) [126,134], freeze-drying of water–ethanol [128,135,136] or aqueous solution [127,128,137,138,139], electrospinning [123,124], co-precipitation in aqueous [73,140,141,142], ethanolic–aqueous [122,126,128,129,130], ethanolic [143], or aqueous–isopropanol solution [131,132], microwave radiation [126,144], evaporation of ethanolic–aqueous [20] and methanolic–aqueous solution [127], complexation in ethanolic–aqueous [129,130,145] or aqueous solution [73,139,141], and sonication [144]. The VE analogues most often formed complexes with βCD [20,106,126,131,135,140,144,145] or its modified derivatives, such as MβCD [138,143], DMβCD [106,134,141], HPβCD [20,127], HEβCD [106], ODSβCD [146], OSAβCD [132], GluβCD [73], HPGβCD [122], and βCD amine derivatives [128]. Less frequently, other native or modified CDs, such as αCD [20], γCD [20,130,139], HPγCD [127], or LRCD [133,147,148] were used.

Most often, CDs and their derivatives were used for complexation as pure substances; however, some researchers created complexes of VE derivatives with, e.g., βCD-functionalized PCL nanofibers [124], HPβCD nanofibrous webs [123], HA-grafted βCD copolymers [136], βCD hydrogel nanocomposites (βCDHGNCs) [142], βCD-loaded sodium caseinate-coated nanoliposomes [135], or βCD/chitosan nanoparticles [147]. Usually, the binary complexes of VE and CDs were investigated, but in the literature, there is also a case of the ternary complexes of VE/βCD with morin in work on hepatoprotection against arsenic toxicity agents [137].

#### 3.3.2. Methods of Analysis of Inclusion Complexes with VE Analogues

In order to elucidate the structures and properties of the obtained inclusion complexes of VE and its analogues and CDs, various methods were used. The most commonly utilized analytical methods in the research of VE/CD complexes include spectroscopic techniques, namely UV-Vis, FTIR, NMR, and PXRD; thermal analysis techniques, such as DSC and TGA; microscopic methods for surface morphology and internal structures at atomic resolution studies, namely SEM and TEM; dispersive light scattering techniques, i.e., DLS, chromatographic techniques (HPLC); and computational methods, such as molecular mechanics (MM), and based on this—molecular docking (Mdoc) and molecular dynamics (MD). Comprehensive information about these methods, and about the features of CD inclusion complexes that can be acquire by them are described in detail in Section 3.1.2. This subsection will focus only on the distinct examples of the application of these techniques in the study of VE/CD complexes.

In Jiao and co-workers’ studies, the differences in the UV spectra of the VE and VE/βCD inclusion complex were examined in order to prove the complexation. The peak of maximal absorption for the K absorption band (resulting from a π-π* transition in a molecule having a conjugated π-π* system) occurred at 284 nm. The authors found that the addition of βCD changed the highest absorption peak to 256 nm, indicating that the extended conjugated chain entered the hydrophobic cavity of βCD, therefore reducing the polarity of the VE microenvironment and enhancing the energy of the л-л* transition [144]. Other authors used UV-Vis spectroscopy in order to determine the encapsulation efficiency of VE by βCD complex-loaded LPs and to assess the amount of released VE [135]. Braithwaite and co-workers utilized UV-Vis spectroscopy for the quantitative determination of VE (and VD3 and VC) contained in dissolved inclusion complexes with various CDs (αCD, βCD, γCD, and HPβCD). In order to remove free VE residue from the outside of each complex, acetone as the organic ‘washing’ solvent was chosen because it only dissolved the uncomplexed VE. Then, the absorbance values were obtained for each inclusion complex solution at the UV max wavelength for VE (295 nm). The inclusion efficiency (IE%) was then calculated using previously plotted standard curves. Overall results indicated that VE/βCD achieved a maximum value of 20.44% IE%, which was a lower result compared to VD3 and VC [20]. In Zhang et al.’s [128] research on VE complexes with βCD amine derivatives, UV-Vis spectroscopy was applied to acquire information about the inclusion proportion of the host and VE by the Job’s method. Under the conditions of a constant concentration of VE, the continuous variation method of the host concentration was applied, and absorbance was monitored by UV-Vis spectroscopy. The inclusion ratios of the inclusion complexes and formation constants (K) were determined using the Benesi–Hildebrand equation. The Benesi–Hildebrand equilibrium indicated that the inclusion ratio between βCD amine derivatives and VE is either 2:1 or 1:1, depending on the type of βCD amine derivative used. The absorption intensity at 292 nm increased with the elongation of the chain at the 6-position of βCD, due to the VE located in the cavity of a more solubilized environment than the hydrophilic condition outside the cavity. These results confirmed that (1) VE was self-assembled into the hydrophobic cavity of βCD and βCD amino derivatives, and (2) an increase in the molecular weight of PET (poly(propylene glycol) bis (2-aminopropyl ether)) at the 6-position of βCD correlates with a deeper penetration of VE in the cavity [128].

The FTIR method was used by many authors in order to determine the VE interactions within solid CD inclusion complexes. The characteristic absorption bands of the VE molecule were in the spectral region where CD absorption is limited, which allowed the detection of these interactions [144]. In the analysis conducted by Koontz et al. [140], the spectra of pure VE exhibited strong bands at 2924 and 2867 cm^−1^, corresponding to asymmetrical methylene and symmetrical methyl stretching vibrations, respectively. These two prominent bands were clearly observable in the physical mixture of VE and βCD. However, these bands were no longer apparent upon complexation within βCD. The characteristic absorption bands of VE at 1460 cm^−1^, associated with the phenyl skeletal vibrations and the overlap of asymmetrical methyl bending and methylene scissoring, as well as the band at 1377 cm^−1^ for symmetrical methyl bending, likewise were absent in the spectra of the VE/βCD complex. It was suggested that a tight fitting of VE within the βCD cavity would hinder these molecular vibrations, hence reducing the intensities of their absorption bands. The spectra of the VE/βCD complex closely resembled that of its βCD host [140]. The FTIR method was also employed to prove the presence of VE in nanofibrous VE/HPβCD inclusion complexes. The spectra of both VE/HPβCD NF and pure HPβCD NF exhibited prominent absorption peaks at around 1020, 1070, and 1150 cm^−1^, which matched to the coupled C–C/C–O stretching vibrations and the stretching vibration of the C–O–C glycosidic bridge in HPβCD molecules. The distinctive FTIR absorption bands of VE at 1377 cm^−1^ belonged to the phenyl skeletal vibrations and the overlap of the asymmetrical methyl bending and methylene scissoring vibrations, while the band at 1458 cm^−1^ was attributed to symmetrical methyl bending. The overlap of the FTIR absorption peaks of HPβCD and VE rendered the FTIR spectra of the VE/HPβCD nanofiber complex rather challenging for the identification of VE. However, the absorption bands at 1377 and 1458 cm^−1^ were observed in a more intense and sharp manner in the case of VE/HPβCD NF, which confirmed the presence of VE guest molecules in the VE/HPβCD NF web samples [123].

Some authors [125,133] applied FTIR in order to confirm the formation of the inclusion complexes between VE or its ester and LRCDs. For this purpose, they compared the spectra of the inclusion complexes, physical mixtures, pure VE, and the LRCDs. No notable differences in the characteristic peak shapes were found for the inclusion complex as compared to the free VE at the principal peaks of 955 (O–H), 1158 (C–O), 1750 (C=O), 2857 (CH_3_), and 2928 cm^−1^ (CH_2_)_n_. Nonetheless, the signal strength of these bands diminished. According to the previous results, the embedding of the guest molecules decreased the movement and signal intensity of the encapsulated molecules. The alternations in the characteristic bands of the guest molecules, such as the decrease in the peak intensity, disappearance, and broadening, were ascribed to the constraints on the tensile vibration of the guest molecules inside the CD cavity. The FTIR spectrum of the LRCD exhibited distinctive bands at 3308 and 1030 cm^−1^, attributed to the stretching of O–H and C–O–C, respectively. The inclusion complex was compared with the LRCD, revealing a shift in the OH stretch from 3308 to 3321 cm^−1^, indicating a considerable increase in hydrogen bonding between the LRCD and VE. In addition, the analysis of the characteristic absorption bands in the spectrum of the physical mixture showed that it was a combination of the individual spectra of the VE and LRCD, suggesting that no interaction between the molecules occurred [125]. The FTIR technique was also used to confirm the formation and structural studies of larger and more complex systems, such as ternary inclusion complexes containing two different guests, i.e., VE and morin molecules loaded into chitosan particles [137], complexes of VE with hyaluronic acid-based βCD-grafted copolymers [136], sodium caseinate-coated and VE/βCD inclusion complex-loaded nanoliposomes [135], or swelling βCD–soy soluble polysaccharide-based core–shell bionanocomposite hydrogels loaded with VE [142]. Although recording FTIR spectra does not require complicated sample preparation and is not difficult to perform, the obtained results are usually not very revealing, and the formation of complexes often requires confirmation by additional methods, e.g., solid-state NMR.

The NMR method provides more details about the structure of the formed complexes than FTIR, allowing for the obtainment of exact and accurate information on the conformation of guest molecule and intermolecular interactions between host and guest molecules by detecting small changes in the chemical microenvironment of the nuclei [149]. Among the NMR techniques, ^1^H-^1^H ROESY NMR and ^13^C CP/MAS NMR deserve special attention in studies on complexation. Two-dimensional ^1^H–^1^H ROESY NMR spectroscopy is a powerful tool for elucidating the spatial arrangement of host–guest inclusion complexes, particularly those formed between CDs and VE derivatives. This technique provides through-space correlation signals (via dipolar interactions), allowing direct insight into the proximity between protons of the CD’s cavity and specific regions of the guest molecule. In the case of VE, ROESY cross-peaks are typically observed between the inner protons of CD’s cavity (H3 and H5) and the protons of the chromanol ring and/or the aliphatic side chain of the guest, indicating its partial or complete insertion into the hydrophobic cavity [122,141]. The presence, intensity, and pattern of ROESY cross-peaks offer valuable information about the inclusion depth, orientation, and dynamic behaviour of the complex in solution. Obtained for inclusion complexes, ROESY data support the proposed geometries of the complexes and complement other spectroscopic and physicochemical methods, confirming the non-covalent nature and supramolecular character of these host–guest systems [122,129,130]. Similarly, as the ^1^H–^1^H ROESY NMR technique is useful for the analysis of inclusion complexes in solution, ^13^C CP/MAS NMR spectroscopy offers detailed structural insights into the nature of inclusion complexes in the solid state, complementing solution NMR. Complexation typically leads to noticeable changes in ssNMR spectra concerning the position of ^13^C peaks, chemical shifts, signals broadening, or splitting. A comparative analysis of physical mixtures, uncomplexed compounds, and freeze-dried or co-precipitated complexes allows the identification of specific interaction sites and conformational constraints imposed by encapsulation. SSNMR also provides evidence for changes in molecular mobility and crystallinity, supporting the amorphization or stabilization of the guest molecule within the CD matrix. In studies of Ke et al. [132] on VE complexation by OSAβCD, the two carbon atoms of the βCD molecules exhibited split signal peaks due to the asymmetric crystalline state of the βCD molecules. These resonance signals changed upon the introduction of OSA (octenyl succinic groups), which altered the chemical environment of the carbon atoms of the CD molecules. The splitting of the peaks in the physical mixture disappeared so that they presented as distinct singlets. In turn, the carbon atoms in the inclusion complex displayed a chemical environment similar to that of the OSAβCD molecules (splitting of signals). All the findings suggested that the CD molecules had a more symmetrical shape after the formation of the inclusion complex [132].

Another analytical method that allows for the analysis of complexes of VE with CDs in the solid state is PXRD [20,134,150]. PXRD is widely applied to prove complex formation by comparing the diffraction patterns of pure substrates, their physical mixtures, and complexes, detecting changes in the crystallinity and possible polymorphic transformations. The PXRD technique had been used to confirm the formation and structural studies of simple complexes formed between VE and native CDs or their derivatives, such as OSAβCD or ODSβCD [132,146], as well as larger and more complex systems, such as ternary inclusion complexes containing two different guests, i.e., VE and morin molecules loaded into chitosan particles [137], complexes of VE with hyaluronic acid-based βCD-grafted copolymers [136], VE/βCD-functionalized PCL nanofibers [124], VE/HPβCD nanofibrous webs [123], or swelling βCD–soy soluble polysaccharide based core–shell bionanocomposite hydrogels loaded with VE [142]. In the research of Mondal and co-workers, the PXRD spectra recorded for the nanoparticles composed of chitosan and ternary inclusion complexes of MOR/VE/βCD revealed the loss of the crystalline character of the NPs, as no sharp peak was observed. This indicated that due to the formation of the nanoparticles, the crystallinity of the used compounds was lost. The PXRD value was consistent with the results obtained from DSC studies [137]. Similar conclusions were drawn by Singh et al. [136] based on their research on the complexes of VE with hyaluronic acid-based βCD-grafted copolymers. The disappearance of sharp peaks and the appearance of a broad halo in the PXRD spectra confirmed the amorphous nature of the obtained systems [136].

Many authors of studies on the complexes of VE derivatives with CDs employed thermal analysis techniques, i.e., DSC and TG, in order to prove complex formation, interaction strength between host and guest molecules, the thermal stability of the inclusion system, and inclusion efficiency [20,73,134,135]. Overall, the thermal analysis of the inclusion complexes demonstrated a lack of endothermic peaks for the vitamin, providing definitive proof of its encapsulation or dispersion inside CD’s cavities [151]. Giordano and co-workers [152] proved that a DSC profile of an inclusion complex often yields a flat curve analogous to the corresponding CD. Such results occur when a bioactive molecule is fully encapsulated inside a CD, with non-covalent interactions between the host and guest causing marked differences in DSC data [153]. The absence of a guest endothermic peak is seen as compelling evidence for the creation of amorphous molecules with distinct physicochemical properties [154].

In research conducted by Koontz et al. [140], the DSC technique was utilized to study the thermal behaviour of the VE/βCD inclusion complex obtained by the co-precipitation method. The glass transition temperatures (Tg) of VE, the physical mixing of VE and βCD, and the VE/βCD inclusion complex were ascertained. The βCD hydrate exhibited a wide endothermic peak ranging from around 25 to 160 °C, indicative of water loss. Two overlapping peaks were identified in the VE/βCD complex; yet, the full peak area was used to compute the enthalpy of the measured effect. The βCD dehydration produced an endothermic enthalpic effect, which must be taken into account in the study of solid-state inclusion processes. The dehydration process involved breaking the bonds between the βCD and water and was then followed by the vaporization of the freed water molecules. If the same reaction scheme was applied to βCD in the physical mixture and the inclusion complex with VE, the ΔH_dehyd_ values were equal to 3.3 and 18.8 kJ/mol of H_2_O, respectively. The ΔH_dehyd_ of βCD in the physical mixture was anticipated to be identical to that of βCD due to the absence of a host–guest interaction. Nonetheless, the ΔH_dehyd_ of 18.8 kJ/mol of H_2_O in the VE/βCD inclusion complex exhibited a significant disparity compared to the ΔH_dehyd_ of the free βCD. This increased ΔH_dehyd_ might be the result of some of the water molecules included within the CD cavity forming hydrogen bonds with the guest molecule [140].

The CDs are often used to increase the heat resistance of several compounds. The effect of complexation on the thermal stability of VE was assessed in an experiment by Siro et al. [145]. The DTG curve of the VE/βCD complex was analyzed in relation to the DTG curves of the individual components (VE and βCD). The DTG curve of VE remained flat between 50 and 190 °C, indicating stability up to 190 °C. Its degradation began at 191 °C. The DTG curve of βCD exhibited a single peak ranging from 38 to 127 °C, with a peak temperature of 90 °C. This phase pertained to dehydration, resulting in a 12.7% water loss. No sign of degradation was seen up to 250 °C. This aligned with the literature, which indicated that the decomposition of CDs occurs at around 300 °C [145]. The DTG curve of the VE/βCD complex exhibited a single stage between 35 and 138 °C. This peak was associated with the process of water loss. The DTG peak minimum temperature was 87 °C, with 7.8% of water weight loss. Subsequent to this peak, no more weight loss was observed until 197 °C, at which point the disintegration of the complex started. Thermal stability assessments revealed that both free VE and the VE/βCD complex were stable at temperatures of up to 190 °C. Authors concluded that the complexation process did not increase the heat stability of VE significantly; however, VE was quite heat stable anyway [145].

The SEM technique was often applied to determine the particle size and morphology of the VE/CD complexes, whereas TEM elucidated internal structures at atomic resolution. In studies conducted by Celebioglu and co-workers, the SEM method was used to investigate the morphology of the electrospun nanofibrous web samples containing VE/HPβCD complexes. The examination of the SEM images demonstrated that the authors successfully produced bead-free nanofibers from pure HPβCD and VE/HPβCD complexes at a 1:2 and 1:1 molar ratio using optimized electrospinning parameters. The average fibre diameters (AFDs) of pure HPβCD, VE/HPβCD (1:1), and VE/HPβCD (1:2) electrospun nanofibers were 745 ± 370 nm, 630 ± 285 nm, and 735 ± 345 nm, respectively. The authors noted that the presence of VE resulted in a reduction in solution conductivity, but at the same time, the viscosity of the solution increased. The adequate aggregation and intermolecular interactions among the HPβCD molecules in highly concentrated (160% (*w*/*v*)) water solutions of VE/HPβCD facilitated the consistent production of nanofibers during electrospinning. On the other hand, very few beads were observed in the VE/HPβCD NF (1:1), likely because of the reduced solution conductivity, which could limit the full stretching of the electrospinning jet at all times [123].

In turn, Cao et al. [125] analyzed the surface morphologies of the complexes of VE with LRCDs. The pure LRCD had an irregular, flaky shape. In turn, the physical mixtures revealed spherical particles mixed with LRCD fragments. After the VE was mixed with the LRCD, it was probable that the VE had adhered to the surface of the LRCDs. In turn, on the SEM image of the inclusion complex, the combination of spherical particles and mixed fragments that formed smaller clusters of particles was observed, which indicated the formation of a complex [125].

The SEM technique was also successfully used in studies conducted by Singh et al. [136] on complexes composed of VE and βCD-grafted hyaluronic acid-based copolymers crosslinked with diphenyl carbonate (DPC). It was observed that different ratios of starting materials and the DPC concentration significantly changed the morphological structures of the samples. For example, the sample with a higher concentration of DPC featured larger micrometre-sized particles, whereas samples with a lower content of DPC were characterized by small-sized particles. The lyophilized samples of the inclusion complexes were porous; this porosity, similar to a sponge-like or gel-like structure, was caused by the freeze-drying step. Structure porosity and particle sizes determined by the SEM method had a great influence on VE solubility and release [136].

Theoretical methods were also utilized in research on complexes of VE/CDs. Among the in silico methods used in the analysis of VE complexes is molecular docking and molecular dynamics (MD) simulations based on molecular mechanics (MM) [20,126,148]. The encapsulation of VE into the LRCD containing 26 glucopyranose units (LRCD26) was studied by Kerdpol et al. [147] by means of molecular dynamics (MD) simulations to investigate their host–guest association process and the preferential binding efficiency upon complexation at a low concentration of VE. The MD data indicated that VE spontaneously interacted with LRCD26 within 20 ns, forming a stable inclusion complex from 150 to 400 ns. All six separate simulations revealed that the inclusion complexes could be categorized into two groups: those in which VE was fully and partly encapsulated inside the cavity of LRCD26. The VE of the first group was associated with 13–14 subunits of LRCD26, while the VE in the alternative group was linked to 6–10 subunits of LRCD26. Moreover, VE was surrounded by a one- or two-turn helix of a half-ring of LRCD26, allowing the remaining cavities to form complexation with a larger number of VE molecules. Consequently, LRCD26 could serve as an enhancer for the solubility and stability of VE via inclusion complexation. All-atom molecular dynamics simulations were conducted on VE and LRCD26 to clarify the host–guest relationship and the binding affinity of the VE/LRCD26 inclusion complexes at a minimal concentration (1:1 molar ratio) of partially enclosed VE inside the cavity of LRCD26. In the group in which the VE was completely enclosed inside the LRCD26 cavity, the system exhibited a relatively low molecular mechanics/Poisson–Boltzmann surface area (MM/PBSA) binding free energy (ΔG_bind_) of less than −11 kcal/mol. In turn, in the latter group (with VE partially enclosed inside the LRCD26 cavity), the ΔG_bind_ values were higher, ~ –7 kcal/mol. The average values of the atomic contacts for the first category were mostly higher than those of the remaining systems, aligning with the stronger binding free energy of the VE/LRCD26 inclusion complexes. The authors concluded that the computational results indicated that LRCD26 was appropriate for association with VE, potentially enhancing aqueous solubility and other biological functions, providing valuable information for future in vitro testing [147].

The continuation of computational research on VE complexes with LRCDs was made by Sangkhwasi et al. [148]. In the mentioned studies, MD simulations were employed to investigated the host–guest encapsulation of VE with an LRCD (CD26, containing 26 glucose units) in different molar ratios, i.e., 1:2, 1:4, 1:6, 2:1, 4:1, and 6:1. Various inclusion complexes of VE with LRCD26 were created using computational approaches to investigate their structural and dynamic behaviour in aqueous solution. The experiments were significant since the encapsulation of VE in CDs with appropriate molar ratios could boost its solubility and bioavailability. The MD simulations indicated that LRCD26 could efficiently function as a carrier for an optimal number of two molecules of VE. On the other hand, the investigation of complexes with varying numbers of LRCD26 molecules per one VE unit indicated that the most likely ratio was 2:1, since LRCD26 was found to self-aggregate in systems with higher molar ratios, i.e., 4:1 and 6:1, leading to restricted VE encapsulation [148].

#### 3.3.3. Applications/Aim of Obtaining VE Derivative Inclusion Complexes with CDs

The main goal for preparing the inclusion complexes of the VE derivatives and CDs is to increase the aqueous solubility, which consecutively leads to enhanced bioavailability and therapeutic efficacy [20,73,122,123,124,129,136,141,144,147,148]. Another purpose for complexation with CDs is to improve the resistance of VE derivatives to decomposition under the influence of light, oxygen, and temperature [20,73,123,124,125,126,127,130,131,132,135,144,147]. Some studies are concerned with the development of a new formulation composed of CD complexes with VE derivatives with a better loading capacity, modified release, and increased absorption [134,135,137,139,142,144], whereas others deal with the application of CDs for the better separation of VE analogues or their metabolites [106,155]. An interesting direction of research on VE complexes with CDs concerns their application in veterinary practice for protective effects on semen or sperm during cryopreservation [138,143]. All publications on VE derivative complexation with CDs are summarized in Table S3 (in Supplementary Materials), and the most interesting applications of VE derivative inclusion complexes with CDs are presented below in more detail.

Liposomes and CDs are often applied in human and veterinary medicine as encapsulating agents to safeguard particularly unstable additives. It is well known that VE can be encapsulated in liposomes or CDs without the need for chemical treatment. Nonetheless, while the encapsulating membrane improves the stability of VE during production and storage, it may also alter absorption and kinetic characteristics (e.g., half-life and bioavailability). The objective of the study lead by Bontempo et al. [156] was to evaluate the comparative bioavailability of one non-encapsulated (oil-based preparation) and two encapsulated (liposome and cyclodextrin) formulations of VE using a plasma kinetics investigation subsequent to oral administration to heifers. Supplementation with VE encapsulated in liposomes showed better results than with VE in inclusion complexes with βCD. The reduced K_d_ for VE encapsulated in liposomes indicated that this formulation could lead to the prolonged retention of VE in plasma compared to the oil-based and βCD preparations [156]. Other studies on the applications of VE complexes with CD in veterinary practice aimed to examine the impact of varying doses of the VE/MβCD complex on cryopreserved ram sperm [138,143] or to assess the possible advantages of VE and CHL when preloaded in CDs, either alone or in combination, to safeguard ram epididymal sperm during the freezing–thawing process [143].

The VE complexes have often been used in more advanced sustained release systems, consisting either of functionalized CD derivatives or containing various additional fibres and polymers. In the study conducted by Celebioglu et al. [123], the inclusion complexes formed between HPβCD and VE were constructed utilizing two distinct molar ratios (VE/HPβCD; 1:2 and 1:1), corresponding to the theoretical loading values of approximately 13% (*w*/*w*) and 26% (*w*/*w*) of VE in the nanofiber (NF) matrix. Following electrospinning and storage, a substantial loading of VE (about 11% *w*/*w* relative to the NF matrix) was maintained in the VE/HPβCD inclusion complexes’ NF. The CD inclusion complexation resulted in a negligible weight loss of approximately 2% *w*/*w*. Although pure VE is water-insoluble, the VE/HPβCD NF exhibited rapid dissolution characteristics. The significantly improved water solubility of VE resulted in the VE/HPβCD NF exhibiting effective antioxidant activity. Furthermore, the VE/HPβCD NF had conferred improved photostability to the sensitive VE through inclusion complexation, allowing the VE/HPβCD NF to maintain its antioxidant activity despite UV-light exposure. Furthermore, a 3-year-old VE/HPβCD NF sample exhibited comparable antioxidant efficacy to freshly generated VE/HPβCD NF, demonstrating the long-term stability of such systems. Those findings indicated that polymer-free electrospun VE/HPβCD NF may possess significant uses in the food, pharmaceutical, and healthcare sectors due to their effective antioxidant properties, increased water solubility, extended shelf life, and superior photostability of VE [123].

A hyperbranched polyglycerol-modified βCD (HPGβCD) was evaluated as a solubilizing agent for VE. The HPGβCD was synthesized using the anionic polymerization of glycidol in the presence of βCD. The HPGβCD enhanced the solubility of VE in comparison to unmodified HPβCD and HPG. The complexation efficacy for HPGβCD was 84 and 7 times superior to that of HPβCD and HPG, respectively. Two-dimensional ROESY NMR results unequivocally demonstrated that VE was encased within the βCD cavity of HPGβCD, exhibiting intermolecular interactions with the external HPG moiety. The results indicated that the augmentation of solubilization was attributable to both inclusion complexation between βCD and VE and hydrotropic solubilization facilitated by branching HPG molecules. Consequently, the HPGβCD was suggested as an excellent choice for solubilizing aliphatic molecules, such as VE [122].

Polycaprolactone (PCL) nanofibers (NFs) encapsulating the VE/βCD inclusion complexes (VE/βCD), characterized by significant antioxidant activity and photostability, were synthesized using electrospinning (PCL/VE/βCD NF). The synthesis of VE/βCD was validated by PXRD. Phase-solubility investigations indicated the development of An-type complexes between VE and βCD. SEM demonstrated the successful production of bead-free nanofibers in the PCL/VE/βCD system. PCL nanofibers encapsulating VE without complexation by βCD were also created for comparative purposes (PCL/VE NF). The antioxidant test results indicated that PCL/VE/βCD NF exhibited superior antioxidant activity relative to PCL/VE NF in a methanol: water (1:1) environment, attributable to the enhanced stability and solubility of αT when in the form of inclusion complexes with βCD. PCL/VE/βCD NF exhibited greater stability against UV radiation compared to PCL/VE NF, attributable to the presence of inclusion complexation. The PCL/VE/βCD NF, characterized by its nanofibrous structure and capacity to encapsulate, presents a unique method for the administration of VE, owing to its enhanced antioxidant activity and superior UV–light stability [124].

**Figure 11:**
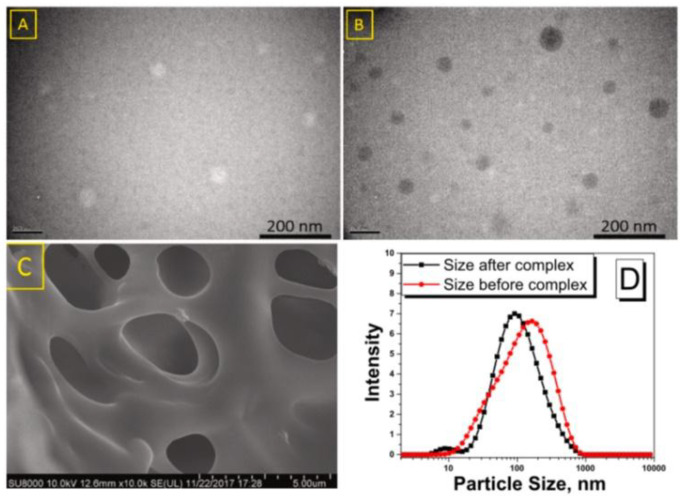
(**A**) Typical TEM image of the HGNPS before the drug loading (transparent particles); (**B**) typical TEM image of the HGNPS after drug loading (dark particles); (**C**) SEM image of the hydrogel with magnification (×10.0 k); (**D**) typical intensity distribution of the HGNPs at 25 °C in PBS before and after drug complex. Reprinted with permission from [142].

**Figure 12:**
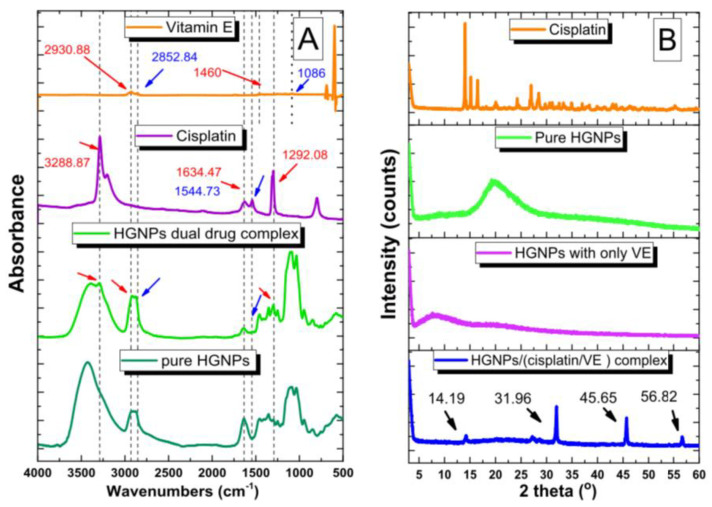
(**A**) FTIR spectra of the VE, pure CDDP, pure HGNPs, and HGNPs complexed with both drugs (VE and pure CDDP). (**B**) PXRD for CDDP, pure HGNPs, and complexed HGNPs with VE and CDDP. Reprinted with permission from [142].

Cross-linked hydrogel nanoparticles incorporating CDs have garnered significant attention in biomedical research due to their unique structural and functional properties. These nanogels combine the hydrophilic, three-dimensional network of hydrogels with the inclusion complexation capabilities of CDs, resulting in versatile platforms for various applications [157]. Chemically cross-linked hydrogel nanoparticles (HGNPs) usually have superior characteristics compared to their physically cross-linked equivalents, especially when used as drug delivery systems. Eid et al. [142] utilized pH-thermal dual-responsive bio-adhesive HGNPs for the dual complexation of cisplatin and VE in order to alleviate cisplatin-induced toxicity due to the reversal of oxidative/nitrosative stress, the suppression of inflammation, and the reduction in total platinum accumulation. The cisplatin was loaded into the HGNPs via chemical conjugation with the carboxyl groups in the HGNP surface by soy polysaccharides (SSPSs), and the host–guest interaction complexed the VE via βCD, thereby enhancing the regulated release and bioavailability of VE [142]. The HGNPs exhibited a consistent size distribution of 90.77 ± 14.77 nm and 81.425 ± 13.21 nm prior to and following the complexation, respectively (Figure 11). The FTIR, PXRD, and zeta potential analyses validated the conjugation (Figure 12). Furthermore, VE exhibited a slower release from the HGNPs at 25 °C compared to 37 °C and 42 °C. The VE-loaded HGNPs demonstrated a prolonged circulation time in vivo compared to the free VE solution.

The development of a novel delivery system for β-carotene (BC) using Pickering emulsions utilizing inclusion complexes VE/βCD combined with soybean lecithin (LC) as a stabilizer of nanoparticles with BC was explored by Hao et al. [158]. Pickering emulsion is an emulsion that is stabilized by solid particles that adsorb onto the interface between the water and oil phases. The authors used LC to substitute βCD hydroxyl groups with hydrophobic fatty chains, resulting in an amphiphilic compound (LC/βCD). Subsequently, VE was encapsulated within the modified βCD complexes, leading to the formation of novel Pickering emulsions stabilized by LC/βCD and LC/βCD/VE complexes for the administration of BC. The surface tension, contact angle, zeta potential, and particle size were determined to evaluate the alterations in complex nanoparticles at different pH levels. Moreover, it was shown that LC/βCD/VE exhibits greater potential as a Pickering emulsion stabilizer compared to LC/βCD due to its reduced particle size (271.11 nm), optimal contact angle (58.02°), and diminished surface tension (42.49 mN/m). The interactions among βCD, soybean lecithin, and VE were studied by FTIR, PXRD, NMR, and TGA. The stability of Pickering emulsions was assessed at different oil-phase volume fractions and nanoparticle concentrations. In comparison to the emulsion stabilized by LC/βCD, the emulsion stabilized by LC/βCD/VE exhibited enhanced storage stability. Furthermore, the delivery of BC indicated that Pickering emulsions stabilized by LC/βCD and LC/βCD/VE could surpass bulk oil and Tween 80-stabilized emulsions regarding UV light stability, storage stability, and bio-accessibility. This research provided novel insights into stabilizer alternatives for Pickering emulsion delivery systems.

Another example of the use of nanocarriers containing CD complexes to improve the solubility and stability of fat-soluble vitamins is a study performed by Souri et al. [135], which aimed to load a βCD/VE inclusion complex into liposomes (LPs) and to coat the created nanocarrier with sodium caseinate (SC) [135]. Three concentrations of SC (2, 4, and 6 mg/mL) were employed for the coating of LPs. The optimal concentration of SC was determined to be 6 mg/mL based on an analysis of particle size, zeta potential, and shape. The zeta potential and particle size of the coated complex-loaded liposomes were +17.90 ± 0.80 mV and 173.9 ± 42.4 nm, respectively. The TEM analysis of the coated complex-loaded liposomes revealed a spherical core–shell architecture. The encapsulation effectiveness of VE in the LPs, as a βCD/VE inclusion complex, was 83.8 ± 3.7%. The establishment of hydrogen-bonding connections among the complex, LPs, and SC was validated using FTIR spectroscopy. The use of SC to coat LPs markedly enhanced their thermal stability. Moreover, the coated LPs exhibited a better-sustained release profile than the uncoated LPs in the simulated gastrointestinal conditions. In conclusion, the engineered nanocarrier exhibited significant advantages and potential applications in the nutraceutical and medical domains.

### 3.4. Vitamin K and Its Derivative Complexes with Cyclodextrins

#### 3.4.1. Preparation Methods of Inclusion Complexes with VK Derivatives

In the scientific papers published so far, the complexes of VK and its derivatives with CDs were practically always prepared in a molar ratio of 1:1 [43,159,160,161,162,163,164]; only Okada et al. [73] used three CD molecules per one VK molecule [73], and Kuboyama et al. applied an excess of VK in relation to CDs, e.g., 2:1 [165].

The main methods for obtaining complexes of VK and its analogues with CDs include the following: kneading with a small amount of solvent (ethanol) [159], co-precipitation in aqueous [43,73,159] or aqueous–ethanolic solution [159], and complexation in aqueous [160,161,162,164] or aqueous–ethylene glycol solution [165]. The VK analogues most often formed complexes with βCD [160,162,163,165] or its modified derivatives, such as MβCD [162], DMβCD [43], HPβCD [162,163], SBEβCD [163], and GluβCD [73]. Less frequently, other native or modified CDs, such as αCD [162], γCD [159,161,165], HPαCD or HPγCD [162], and AMDCDs [164] were used.

Native CDs and their derivatives were used for complexation as pure substances (without any further functionalization), and unlike with other fat-soluble vitamins, only binary complexes in the case of VK were reported.

#### 3.4.2. Methods of Analysis of Inclusion Complexes with VK Analogues

In order to investigate the structures and features of the acquired inclusion complexes of VK and its derivatives with CDs, various methods were used. The most commonly employed analytical methods in the research of VKCD complexes include spectroscopic techniques: UV-Vis, FTIR, PXRD, and NMR; thermal analysis techniques, such as DSC and TGA; TEM; and QM calculations. Common information about these methods, and about the structure details of CD inclusion complexes that can be obtained by them, is reported in Section 3.1.2. This subsection will focus only on the distinct examples of the application of these in the study of VK/CD complexes presented in scientific papers.

The UV-Vis spectroscopy is a crucial method for examining the inclusion effect between CDs and small compounds such as VK derivatives. The formation of a specific inclusion complex between the guest and the CD host results in alterations in intensity, peak shape, or the absorbance position of the maximum peak [166].

In the study by Szejtli et al. [43], the UV-Vis spectroscopy was utilized to analyze the structures of the complexes of VK3 and DMβCD and the supramolecular interactions between them. The UV-Vis spectra in aqueous solutions, regardless of the presence or absence of DMβCD, were substantially comparable. The slight difference was that the first maximum occurred at 335 nm (instead of at 340 nm), suggesting that the benzene ring of the naphthoquinone molecule was positioned inside the apolar DMβCD cavity. The quinonoid segment of the spectrum resembled that of menadione in aqueous solution, indicating that the quinone moiety of the molecule was located outside of the DMβCD ring. Upon UV irradiation, this system underwent fast alteration, resulting in the formation of an entirely new spectrum within five minutes. All the characteristic λ_max_ values of an aqueous VK3 solution were reduced, and a single intensive λ_max_ at 228 nm could be observed. This intensive maximum could be ascribed to the VK3 dimers of types “A” and “B”. Consequently, in an aqueous solution containing DMβCD, additional products were likely generated, since peaks at 213, 228, and 243 nm were observed. Based on these results, the authors concluded that the presence of DMβCD did not protect VK3 from UV light-induced decomposition. The observation that, in aqueous or DMβCD containing aqueous solutions, different products were formed on UV irradiation, suggested an interaction between the VK3 and DMβCD molecules [43].

Li et al. [164] applied UV-Vis spectroscopy to assess the VK3 loading in inclusion complexes with synthesized novel amphiphilic cyclodextrin (AMCDs), with modification of positively charged imidazolium cation groups and alkyl chains at the lower rim of the CD ring. Owing to their amphiphilic characteristics, these AMCDs were capable of self-assembly and co-assembly with hydrophobic drugs in an aqueous environment. The objective of the study was to develop a VK3 carrier with AMCD to address the VK deficit in haemodialysis patients during the heparin neutralization process. The calibration curve indicated a linear correlation between the absorbance of VK3 at 330 nm and VK concentration within the range of 2–22.5 μM. Since the AMCD had no absorption at 330 nm, from the UV-Vis spectrum of VK3/AMCD co-assembly, the concentration of VK3 was readily determined using the calibration curve. The VK3 loading efficiency and the encapsulation efficiency were determined as 6% and 40%, respectively. Following the morphological transformation seen by TEM and DLS after the capture of heparin, about 64% VK3 was released, as determined from the UV-Vis absorption spectrum [164].

NMR spectroscopy often serves as the additional confirmation of assumptions concerning the inclusion of VK inside the CD cavity based on the UV-Vis spectroscopy and thermal analysis methods and provides detailed information about complex structure and supramolecular interactions [43]. In the research led by Zielenkiewicz, ^1^H NMR was used for the verification of the formation of complexes between VK3 and various CDs. An addition of the variable amounts of HPαCD, βCD, HPβCD, MβCD, and HPγCD induced the downfield shift in the signals of all VK3 ^1^H resonances. The conversion of the dependence of proton chemical shift changes (Δδ) induced by the complex formation of VK3 with a reciprocal Benesi–Hildebrand plot produced linear relationships that corroborated the formation of the 1:1 complexes. The comparison of Δδ values highlighted that the VK3 proton signals underwent the most significant alterations upon binding with HPαCD. The significant alteration of the signals of all the VK3 protons caused by the introduction of αCD was also noted. In the case of complex formation with all other CDs, the maximal Δδ values were observed, but only for certain protons. This difference could be explained by the distinct binding mechanism of VK3 with CDs. VK3 likely fitted tightly inside the αCD cavity and progressively more loosely in βCD and γCD. The elevated Δδ values for the H7, H8, and H3 protons, with diminished Δδ values for the H6, H9, and H11 protons, seen during the complexation of VK3 with βCD, indicated the production of two distinct types of inclusion complexes in an aqueous solution. In one of them, the aromatic ring had no methyl group incorporated into the hydrophobic cavity, whereas in the second one, on the contrary, the ring containing a CH3 group was inserted into the cavity. The incorporation of the VK3 molecule was limited due to the steric barrier posed by the ketonic groups at the one and four locations. Furthermore, HPβCD and MβCD, relative to unmodified βCD, induced the more significant chemical shift changes in the VK3 protons. It was ascribed to the profound incorporation of VK3 into the cavity of substituted βCDs and aligned with the conclusions derived from the thermodynamic parameters of complex formation. In the case of a complex with HPγCD, the Δδ values for the H7, H8, and H11 protons were the highest, while the Δδ for the H6, H9, and H3 protons was almost zero. It indicated that HPγCD produced two types of complexes with VK3; nevertheless, the penetration of the VK3 molecule into the HPγCD cavity was shallow [162].

DSC is a thermal analysis technique that is used to measure the heat capacity (Cp) of materials along with the temperature change. A crystal material displays a melting or crystallization event associated with an enthalpy change. As the melting temperature (Tm) is reached, an endothermic peak appears, related to the melting transition on the DSC curve. Amorphous materials do not have an organized solid state, hence during heating, they exhibit a second-order phase change in which the heat capacity abruptly changes, and this transition from the glassy to rubbery state is called the glass transition. This transition appears as a broad shift in the baseline of the DSC curve, and Tg is called the glass transition temperature.

Therefore, the DSC technique is often utilized in the studies of VK complexes with CDs. The second thermal method of analysis used for studying the VK3 complexes with CDs is TGA. This technique allows for the characterization of mass loss profiles, decomposition temperatures, and thermal behaviour differences between pure components and the resulting complexes, proving their formation and providing insights into their stability [73,159].

#### 3.4.3. Applications/Aim of Obtaining VK Derivative Inclusion Complexes with CDs

The general target for preparation the inclusion complexes of VK derivatives and CDs is to improve the aqueous solubility, which results in increased bioavailability and medicinal effectiveness [43,73,162,164]. Another reason for complexation with CDs is to enhance the resistance of VK analogues to decomposition under the influence of light, oxygen, and temperature [43,73,159,165]. Some studies are concerned with developing a new formulation consisting of CD complexes with VK analogues with a better loading capacity or modified release [159,164], whereas others deal with improving the luminescence and fluorescence detection of VK [160,163]. An interesting direction of research on VK complexes with CDs concerns their application in veterinary practice [159]. All publications on VK complexation with CDs are summarized in Table S4 (in Supplementary Materials), and the most interesting applications of VK derivative inclusion complexes with CDs are presented below in more detail.

Balancing and neutralizing the heparin dose post-surgery and hemodialysis is crucial in medical and therapeutic practice. In work conducted by Li et al. [164], the design and synthesis of a series of novel amphiphilic multicharged cyclodextrins (AMCDs) for use as anti-heparin coagulants was made. The AMCD assembly demonstrated selective heparin binding through multivalency and exhibited superior neutralization effects against both unfractionated heparin and low-molecular-weight heparin compared to protamine in plasma. Simultaneously, the co-assembly of AMCD and VK was developed to achieve heparin-responsive VK release and offer an innovative treatment for VK deficiency in hemodialysis patients. The VK/AMCD co-assembly for heparin neutralization and VK replenishment presents a viable possibility for therapeutic anti-heparin coagulation [164].

A promising theranostic nanosystem, VK3/CPT/RuCD, was engineered through the host–guest driven self-assembly of fluorescent adamantine-functionalized Ru (II) complexes and ROS–labile cyclodextrin-modified thioketal linkers, facilitating the effective co-encapsulation of the anticancer drug camptothecin (CPT) and VK3. Due to the synergistic interaction between the intracellular redox cycling of VK3 and the significant ROS-induced degradation of nanoparticles, VK3/CPT/RuCD promoted cancer-specific ROS amplification and targeted drug release within cancer cells, thereby achieving selective tumour eradication with minimal side effects both in vitro and in vivo, demonstrating a more pronounced therapeutic effect than free anticancer agents. The menadione structure of encapsulated VK3 effectively quenched the inherent fluorescence of RuCD, resulting in a fluorescence enhancement phenomenon observed alongside ROS-triggered drug release, which can be employed for the real-time monitoring of drug release both in vitro and in vivo [167].

The interactions in the complexes formed between VK3 and βCD, HPβCD, and SBEβCD were investigated by steady-state fluorescence measurements. The several aspects influencing the inclusion process were analyzed comprehensively. The formation constants and inclusion stoichiometry for VK3/CDs were established. The findings indicated that the inclusion capacity of βCD and its derivatives ranked as follows: SBEβCD > HPβCD > βCD. The associated inclusion mechanism was suggested to elucidate the inclusion process. A method for quantifying VK3 was developed, exhibiting a linear range of 2.5 × 10^−6^ to 5.0 × 10^−4^ M, and was employed to analyze VK3 tablets. The developed fluorescence-based method offered a reliable approach for quantifying VK3, which could be beneficial for quality assurance in the pharmaceutical industry [163].

The intricate interactions between native CDs (αCD, βCD, γCD) and a modified dimethyl derivative (DMβCD) and quinones (three 9,10-anthraquinone sulphonates and two 1,4-naphthoquinones) in a water–ethylene glycol 1:1 mixture were examined utilizing the spectroscopic methods. Phosphorescence spectroscopy at low temperatures (77 K) provided detailed insights into the electronic states and interactions within the complexes. The findings suggested that the structures of the inclusion complexes vary depending on the specific combination of cyclodextrin and quinone used, e.g., VK3 was observed to efficiently produce the photodimer in the presence of γCD. Understanding the formation and properties of CD–quinone inclusion complexes is essential for applications in areas such as photochemistry, molecular recognition, and the development of novel materials. The ability of cyclodextrins to modify the photophysical behaviour of quinones through inclusion complexation can be leveraged in designing systems for controlled photoreactions and in stabilizing reactive species [165].

Studies conducted by Tabushi et al. [168] explored the catalytic role of βCD in synthesizing the analogues of vitamins K1 and K1 through a one-step process. βCD acted as a catalyst by encapsulating specific reactants within its cavity, thereby influencing the reaction’s efficiency. The research demonstrated that the presence of βCD enabled the direct and efficient synthesis of VK analogues in a single step. This method contrasted with traditional multi-step synthetic routes, offering a more streamlined approach to producing these compounds. Electrophilic allylation at the C3 position of 2-methylhydronaphthoquinone-1,4 with allyl, crotyl, methallyl, or prenyl bromide was effectively established, yielding a highly selective one-step synthesis of the corresponding vitamin VK1 (or VK2) analogues, utilizing βCD in a dilute aqueous alkaline solution. To clarify the foundation of this intriguing inclusion catalysis, akin to “ligase and/or oxidase” reactions, mechanistic investigations were conducted. The precise inclusion binding of the substrate, 2-methylhydronaphthoquinone, by βCD (K_a_ = 490 M^−1^, pH 3.55) promoted proton dissociation, leading to a reduction in its pKa value from 9.45 for the uncomplexed form to 8.9 for the complexed form. The kinetic results indicated that the allylation rate of αnaphthol at a pH of 10.4 was augmented by 2.5 to 3.5 times in the presence of βCD, leading to the conclusion that the nucleophilic reactivity of the partially charged carbanion was enhanced within the hydrophobic cavity, thereby accelerating the allylation reactions facilitated by βCD. An additional significant element of the procedure was the intriguing observation that the 2-methylnaphthosemiquinone anion radical (identified by ESR), generated from the oxidation of allylated hydronaphthoquinone by molecular oxygen, exhibited a remarkable affinity for βCD. This suggested that oxidation could primarily occur via the complexed form of the allylated hydroquinone or that the inclusion binding extended the lifespan of the semiquinone anion radical. Several VK analogues were identified as compounds that exhibited significant vulnerability to oxidative degradation caused by hydrogen peroxide, a byproduct of the oxidation process. βCD efficiently safeguarded the quinones from hydrogen peroxide assault, resulting in oxidation rates ranging from 1/9 to 1/17 of those observed for the uncomplexed quinone [168].

The domain of cyclodextrin chemistry is advancing swiftly, emphasizing the highly selective production of bioactive molecules by inclusion catalysis. For example, the successful utilization of CDs in the one-step synthesis of VK2 analogues in a dilute aqueous alkaline solution was described. VK1 was initially produced from 2-methylhydronaphthoquinone-1,4 and phytol by the Friedel–Crafts reaction. Nonetheless, this method frequently yielded unwanted compounds due to alkylation at the C2 position and subsequent cyclization to naphthotocopherol. The cited work presented a new synthesis method of VK1 or VK2 analogues, utilizing βCD. The yield of the VK1 or VK2 analogues produced by the inclusion complex was significantly greater than under standard conditions, suggesting that βCD played a crucial role in the allylation process. The pronounced catalytic activity of CD appeared to stem from the enhanced nucleophilicity of the carbon atom in the naphthohydroquinone monoanion, encapsulated within the CD cavity [169].

Fat-soluble vitamins, characterized by low polarity and suitable molecular size, can establish stable (K > 10^3^ mol^−1^) inclusion complexes with various cyclodextrins through interactions with their hydrophobic cavities. This review indicates that these complexes have been synthesized and analyzed extensively for over 40 years, with a continuous increase in the volume of related publications.

Multiple analytical methods are frequently used for the characterization of these complexes, including spectroscopic (NMR, UV-Vis, Raman, IR), thermal (TGA and DSC), diffraction-based (PXRD and SCXRD), and microscopic (SEM and TEM) approaches [170]. This large variety of methods arises from their complementary character in providing valuable data on the properties and dynamics of complexes. Those experimental works are sometimes supported by the molecular modelling results, performed at both the molecular mechanics and quantum mechanics levels. In addition to structural and physicochemical investigations aimed at determining the complex ratio, stability, and CD–vitamin interactions, numerous application-oriented studies have also been published. CDs have effectively served as solubilizers and absorption enhancers in pharmaceutical formulations across multiple administration routes or even as catalysts of many diversified biochemical processes. Nonetheless, the variety of both native and substituted CDs indicates substantial potential for more investigation in this domain. Therefore, we expect that this study will facilitate the planning of similar research and allow for the comparison of newly obtained results with previously published data.

## Figures and Tables

**Table 1:** Overview of the five vitamin D variants.

Vitamin Name	Chemical Composition
Vitamin D_1_	1:1 mixture of ergocalciferol and lumisterol
Vitamin D_2_	ergocalciferol (derived from ergosterol)
Vitamin D_3_	cholecalciferol (synthesized from 7-dehydrocholesterol in the dermis)
Vitamin D_4_	22-dihydroergocalciferol
Vitamin D_5_	sitocalciferol (derived from 7-dehydrositosterol)

## Data Availability

Not applicable.

## Supplementary Materials

The following supporting information can be downloaded at: https://www.mdpi.com/article/10.3390/ijms26136110/s1.

## Abbreviations

The following abbreviations are used in this manuscript:

2HPβCD	2-hydroxypropyl-β-cyclodextrin
7-DHC	7-dehydrocholesterol (provitamin VD3 precursor)
AFM	atomic force microscopy
AFS	atomic fluorescent spectroscopy
AMCDs	amphiphilic multicharged cyclodextrins
AMF	atomic force microscopy
BC	β-carotene
CDs	cyclodextrins
Chol-βCD-Ac	cholesteryl-acetyl-βCD
CirD	circular dichroism
CLSM	confocal laser scanning microscopy
CMβCD	carboxymethylβcyclodextrin
COSY	correlation spectroscopy
CP/MAS	cross-polarization magic angle spinning
DFT	density functional theory
DLS	dynamic light scattering
DMβCD	dimethyl-βCD
DMγCD	dimethyl-γCD
DSC	differential scanning calorimetry
DPC	diphenyl carbonate
DTG	derivative thermogravimetric analysis
EγCD	ethyl-γCD
EPI	Epiclon (5-(2,5-dioxotetrahydrofuran-3-yl)-3-methyl-3-cyclohexene-1,2-dicarboxylic anhydride)
FLS	fluorescence spectroscopy
FTIR	Fourier-transform infrared spectroscopy
GalαCD	galactosyl-αCD
GluαCD	glucosyl-αCD
GluβCD	glucosyl-βCD
HA	hyaluronic acid
HF	Hartree–Fock
HEβCD	hydroxyethyl-βCD
HMDI	hexamethylene diisocyanate
HGNCs	hydrogel nanocomposites
HPαCD	hydroxypropyl-αCD
HPβCD	hydroxypropyl-βCD
HPγCD	hydroxypropyl-γCD
HPLC	high performance liquid chromatography
ITC	isothermal titration calorimetry
LDA	laser–Doppler anemometry
LDPE	low-density polyethylene films
LLDPE	linear low-density polyethylene films
LRCDs	large-ring cyclodextrins
LS	light scattering spectroscopy
MβCD	methyl-βCD
MD	maltodextrin
MDoc	molecular docking
MEKC	micellar electrokinetic chromatography
MM	molecular mechanics
MP	Møller–Plesset
MTT	methylthiazolyl diphenyl tetrazolium assay
NFs	nanofibers
NLPs	nanoliposomes
NMR	nuclear magnetic resonance
ODSβCD	octadecenyl succinic- βCD
OγCD	octenyl-γCD
ONIOM	Our N-layered Integrated molecular Orbital and molecular Mechanics
OSAβCD	octenyl succinic-βCD
OTM	optical transmission microscopy
PCL	polycaprolactone
PCS	photon correlation spectroscopy
PEGDGE	poly(ethylene glycol) diglycidyl ether
PI	polydispersity index
PM3	parametric method 3
PMβCD	partially methylated βCD
PS	phosphorescence spectroscopy
PVA	poly(vinyl alcohol)
PXRD	powder X-ray diffraction
QC	quantum mechanics
QM	quantum mechanics
RA	retinoic acid
Raman	Raman spectroscopy
ROESY	rotating-frame Overhauser effect spectroscopy
SAβCD	stearoyl-βCD
SBEβCD	sulfobutyl ether βCD
SCXRD	Single-crystal X-ray diffraction
SDTA	single differential thermal analysis
SEM	scanning electron microscopy
SSPS	soy soluble polysaccharide
SWV	square-wave voltammetry
TGA	thermogravimetric analysis
TMβCD	trimethyl-βCD
TOC3	tocotrienol
UDV	ultradeformable vesicles
UV–Vis	ultraviolet–visible spectroscopy
VE	vitamin E (α-tocopherol)
VK	vitamin K
VD	vitamin D
VA	vitamin A (retinol)
XPS	X-ray photon electron spectroscopy
XRD	X-ray diffraction
