# Supplementary material for: Complexes of Fat-Soluble Vitamins with Cyclodextrins
Source: Int J Mol Sci. 2025 Jun 25;26(13):6110. doi: 10.3390/ijms26136110 (PMC12250296; doi:10.3390/ijms26136110)
Supplement: Supplementary file 1 [file ijms-26-06110-s001.zip › ijms-3634546-supplementary.pdf]

International Journal of Molecular Sciences

MDPI

**Table S1.** Overview of the vitamin A and CD complexes.



| Vitamin or vitamin derivative | CD type | Molar ratio (vitamin:CD) | Preparation method | Analysis method | Aim of study/Application | First author, year of publication [Article No] |
|---|---|---|---|---|---|---|
| VA | EγCD, OγCD | 1:1 | Freeze-drying of water-ethanol solution | DSC, UV-Vis spectroscopy, HPLC | Increase of UV and temperature stability and solubility in water. Enhancement of skin permeation | Sapino 2007 [44] |
| VA | OAβCD, SAβCD | ND | Nanoprecipitation from ethanol solution | TGA, FTIR, DLS, TEM, $^{1}H/^{13}C$ NMR, FLS, MS | Improvement of stability to UV radiation | Kim 2016 [63] |
| VA, VA acetate, VA palmitate | βCD | 1:2 (VA), 1:1 (VA esters) | Freeze-drying of water-ethanol solution | FTIR, $^{1}H$ NMR, SEM, UV-Vis spectroscopy, Mdoc | Enhancement of solubility and stability at elevated temperature | Xu 2021 [39] |
| VA palmitate | βCD | 1:1, 1:2 | Homogenization of VA palmitate suspension in CD's aqueous solution | FLS, UV-Vis spectroscopy | Determination of stoichiometry and association constants of complexes | Palmieri 1992 [46] |
| VA palmitate | αCD, βCD, γCD with addition of MD | ND | Kneading with small amounts of water (15% w/w) in elevated temperature and vacuum drying | UV-Vis spectroscopy, SEM | Enhancement of stability and solubility | Koeda 2014 [53] |

 https://doi.org/10.3390/xxxxx

| | | | | | | |
|---|---|---|---|---|---|---|
| VA palmitate | βCD | 1:1 | Freeze-drying of phosphate buffer solution | DSC, TGA, SDTA, FTIR, UV-Vis spectroscopy | Increase of stability toward temperature, oxygen and UV light and enhancement of solubility in water | Vilanova 2015 [47] |
| VA acetate | HPβCD, HPγCD nanofibrous webs | 1:2 | Electrospinning of CDs aqueous solution mixed with VA acetate powder | SEM, ATR-FTIR, PXRD, DSC, TGA, $^{1}$H NMR, UV-Vis spectroscopy | Preparation of novel fast-dissolving oral formulations with increased water solubility, enhanced bioavailability, stability and antioxidant activity | Celebioglu 2020 [45] |
| VA acetate | βCD functionalized PVA nanofibres | ND | Electrospinning of CDs/PVA aqueous solution mixed with VA acetate powder | PXRD, DSC, XPS, SWV, TGA, SEM | Prolongation of shelf life and increased thermal stability | Lemma 2015 [59] |
| VA propionate | DMβCD | 1:10 | Freeze-drying of aqueous solution | $^{1}$H/ROESY NMR, HPLC, MS | Increase of solubility, stability and skin penetration | Weisse 2002 [52] |
| VA propionate | αCD, βCD, γCD, DMβCD | 1:1, 1:2 | Freeze-drying of aqueous solution | $^{1}$H/ROESY NMR, HPLC, MS | Increase of solubility, stability and skin penetration | Weisse 2004 [57] |
| VA propionate | Chol-βCD-Ac | ND | Vacuum evaporation of acetone-aqueous solution of VA propionate and Chol-βCD-Ac with surfactant (Pluronic® PEF 68) | $^{1}$H/COSY/ROESY NMR, TEM, HPLC, PCS | Enhancement of stability and skin penetration | Weisse 2009 [74] |

| | | | | | | |
|---|---|---|---|---|---|---|
| VA propionate | βCD | 1:2 | In silico | PM3, ONIOM2 | Theoretical assessment of the inclusion complex geometry, stability and conformational changes of VA propionate in CD's cavity | Mallem 2011 [64] |
| retinal | βCD | ND | Evaporation of mixture of retinal's hexane solution and CD's aqueous solution and freeze-drying | FTIR, PXRD $^1H/^{13}C$ NMR, AFM | Enhancement of photochemical stability | Munoz-Botella 1996 [65] |
| RA | HPβCD | 1:1, 1:2 | Homogenization of RA suspension in CD's phosphate buffer solution | HPLC | Improvement of water solubility and photostability | Lin 2000 [71] |
| RA | βCD | 1:1, 1:3 | Freeze-drying | FTIR, $^1H$ NMR, HPLC | Protection against photo-degradation upon exposure to UV radiation and fluorescent light | Caddeo 2007 [49] |
| RA | DMβCD | 1:1 | Co-precipitation, freeze-drying, spray-drying, kneading with small amount of water | FTIR, $^1H$ NMR, DSC, PXRD, AFM, Raman spectroscopy, MM | Determination of stoichiometry and structure | Ascenso 2011 [69] |
| RA | DMβCD | 1:4 | Kneading without solvent | PCS, AFM, LDA, HPLC | Development of novel topical formulation with improved physicochemical parameters, e.g. loading capacity and chemical stability | Ascenso 2013 [51] |
| RA | βCD, DMβCD, HPβCD | 1:1 | Solvent evaporation from mixture of RA's ethanol | HPLC, SEM, FTIR, DSC, PXRD, $^1H$ NMR | Increase of solubility for topical use | Montassier 1997 [66] |

| | | | | | | |
|---|---|---|---|---|---|---|
| | | | solution and CD's water solution | | | |
| RA | βCD, HPβCD | 1:1 | Kneading with small amount of hydroalcoholic solution | FTIR, DSC, MDoc | Improvement of solubility and tissue tolerability during topical use | Fathalla 2024 [54] |
| RA | DMβCD, TMβCD, DMγCD | 1:2 | Slow cooling of a homogenous solution of both components in water-ethanol mixture | UV-Vis spectroscopy | Acceleration of dissolution and increase of solubility for toxicological studies | Pitha 1983 [70] |
| RA | αCD, GalαCD | 1:1, 1:2 | Freeze-drying mixture of water solution of CD and ethanol solution of RA | $^1$H NMR, FTIR, PXRD | Increase of solubility for toxicological studies | Seo 2004 [56] |
| RA | αCD, HPβCD | 1:1, 1:2 | Freeze-drying of aqueous solution | DSC, FTIR, HPLC, PXRD, MDoc | Improvement of water solubility and photostability | Yap 2005 [58] |
| VA, retinal, VA acetate, RA | βCD, HPβCD, DMβCD, TMβCD | 1:1, 1:2 | Solvent evaporation from mixture of hexane or ethanol solution of retinoid and water solution of CD | UV-Vis spectroscopy, $^1$H NMR, HPLC | Limiting photo-isomerization reactions, protection against photo and oxidative degradation | Munoz-Botella 2002 [48] |
| BC | HPβCD | ND | Co-precipitation from mixed aqueous solution of CD and acetone solution of BC | Raman spectroscopy, UV-Vis spectroscopy, HPLC | Protection from light, temperature, and oxygen | Celitan 2021 [61] |

| | | | | | | |
|---|---|---|---|---|---|---|
| BC | βCD, γCD | 1:4 | Kneading with small amount of solvent (water) and freeze-drying | $^{1}$H NMR, DLS, MS | Study of large aggregates formation in aqueous solution | Mele 1998 [50] |
| BC | MβCD, HPβCD, HEβCD | 1:1 | Solvent evaporation after mixing dichloromethane solution of BC and ethanol solution of CD | FTIR, FLS, UV-Vis spectroscopy | Enhancement of water solubility, stability and antioxidant properties of BC | Celik 2017 [72] |
| BC | βCD | 1:1 | Co-precipitation from ethanol-water 25:75 (v/v) ultrasonicated mixture | FTIR, $^{1}$H NMR, TEM | Increase of water solubility and bioavailability | Kaur 2016 [62] |
| BC | βCD-based nanosponges (CDNS) crosslinked with HMDI or EPI (1:4; 1:2 respectively) | ND | Solvent evaporation after mixing acetone solution of BC and water solution of CDNS and freeze-drying | FTIR, SEM, DLS, PXRD, DSC, UV-Vis spectroscopy | Development of BC carrier with improved solubility and stability | Yazdani 2021 [75] |
| BC | magnetic chitosan<br>βCD biopolymer | ND | Extraction and evaporation | SEM, FTIR, TGA, HPLC | Development of ultrasonic-assisted solid phase extraction procedure for BC from vegetables | Dai 2019 [76] |
| BC | βCD, OSβCD | ND | Solvent evaporation after mixing acetone solution | FTIR, PXRD, $^{13}$C CP/MAS NMR, AFM, SEM | Improvement of physical and | Niu 2019 [67] |

| | | | | | | |
|---|---|---|---|---|---|---|
| | | | of BC and water/ethanol solution of CD | | oxidative stability of the BC emulsion for food industry | |
| BC | HPβCD, HPγCD | ND | Electrospinning from aqueous solution of CD and BC | SEM, FTIR, PXRD | Increase of water-solubility and photostability, enhancement of antioxidant bioactivity | Yildiz 2023 [60] |
| VA | βCD | 1:1 | Kneading in mortar with pestle with addition of water | $^1$H NMR | Assessment of competition between bile acids and VA or VD3 in the formation of inclusion complexes for estimation of the risk of depletion of lipophilic vitamins after ingestion of βCD | Comini 1994 [55] |
| VA | αCD, βCD, MβCD, DMβCD, TMβCD, SBEβCD, HPβCD, HPαCD, HPγCD | | Complexation in mixture of ethanol VA solution and PBS solution of CD | FLS, HPLC | Stabilization for ophthalmic purpose | Semenova 2002 [68] |
| VA | GluβCD | 1:2 | Complexation in aqueous solution | DSC, HPLC | Improvment of stability and solubility | Okada, 1990 [73] |

**Table S2.** Overview of the vitamin D and CD complexes.

| **Vitamin or vitamin derivative** | **CD type** | **Molar ratio (vitamin:CD)** | **Preparation method** | **Analysis method** | **Aim of study/Application** | **First author, year of publication [Article No]** |
|---|---|---|---|---|---|---|
| VD3 | βCD | 1:2 | Microwave radiation | SEM, DSC, DTG, TG, FTIR, $^{1}H/^{13}C$ NMR | Enhancement of solubility and stability of VD3 | Bakirova, 2020 [93] |
| VD3 | βCD | 1:2 | Co-precipitation from ethanol-aqueous solution | SEM, IR, $^{13}C$ NMR, DTG, DSC, PXRD | Enhancement of photo and thermal stability | Szejtli, 1980 [42] |
| VD2-VD5, ProVD2-ProVD5 | MβCD | ND | MβCD as mobile phase modifier | HPLC-MS, $^{1}H$ NMR | Improvement of separation of VD2-VD5 and their provitamins | Shimada, 1995 [104] |
| 7-DHC | HPβCD | 1:1 | Freeze-drying | HPLC, FTIR, DSC, UV-Vis spectroscopy | Enhancement of solubility for cosmetic applications | Kim, 2010 [94] |
| VD3 | βCD NLPs | 1:1 | Complexation in aqueous solution | FTIR, SEM | Development of new controlled release for nutritional therapy | Ebrahimi, 2023 [95] |
| VD2, VD3 | GluβCD | 1:2 (VD2), 1:1 (VD3) | Co-precipitation in aqueous solution and vacuum drying | DSC, HPLC | Enhancement of light stability and water solubility | Okada, 1990 [73] |
| VD3 | βCD, γCD, PMβCD | ND | Complexation in J-medium/aqueous solution | HPLC | Acceleration and enhancing of VD3's microbial conversion to active metabolites | Takeda, 1994 [121] |

| | | | | | | |
|---|---|---|---|---|---|---|
| VD2, VD3 | βCD, DMβCD, HEβCD | ND | βCD, DMβCD, HEβCD as mobile phase modifiers | HPLC, MEKC | Improvement of separation of VD derivatives | Spencer,1997 [106] |
| VD2 | βCD | 1:1, 1:2 | Spray-drying, evaporation, kneading | DSC, HPLC PXRD, $^{13}C$ NMR | Enhancement of solubility | Palmieri, 1993 [96] |
| VD3 | βCD | 1:1:5, 1:1:10 (ternary VD3:$Al^{3+}$: βCD complexes) | Evaporation of ethanolic-aqueous solution | PXRD, $^{13}C$ NMR | Development of new controlled release systems | Soares, 2012 [102] |
| PreD3 | αCD, βCD, MβCD | 1:1 | Complexation in ethanolic-aqueous solution | HPLC, UV-Vis spectroscopy | Study of catalytic isomerization of PreD3 to VD3 | Tian, 1995 [97] |
| VD3 | βCDNS | 1:4 | Milling | TGA | Improvement of stability and absorption in gut-brain axis | Uberti, 2023 [100] |
| VD3 | HPβCD | 1:1, 1:2 | Spray drying | MD, DSC, PXRD, FTIR, SEM | Enhancement of solubility | Wang, 2022 [98] |
| VD3 and its hydroxylated metabolites | γCD, MβCD | ND | γCD, MβCD as mobile phase modifiers | HPLC | Development of separation method for VD3 metabolites | Higashi, 2000 [105] |

| | | | | | | |
|---|---|---|---|---|---|---|
| VD3 | αCD, βCD, γCD, HPβCD | 1:2,5, 1:5, 1:10 | Evaporation of ethanolic-aqueous solution | FTIR, DSC, PXRD, UV-Vis spectroscopy, MM | Enhancement of stability and solubility | Braithwaite, 2017 [20] |
| VD3 | βCD | 1:1:5, 1:1:10 (ternary VD3:$Co^{2+}$:βCD, VD3:$Cu^{2+}$:βCD, VD3:$Zn^{2+}$:βCD complexes) | Evaporation of ethanolic-aqueous solution | PXRD, $^{13}C$ NMR, UV-Vis spectroscopy | Development of new controlled release systems | Merce, 2009 [101] |
| VD3 | βCD | 1:1 | Kneading with small addition of water | $^{1}H$ NMR | Assessment of competition between bile acids and VA or VD3 in the formation of inclusion complexes for estimation of the risk of depletion of lipophilic vitamins after ingestion of βCD | Comini, 1994 [55] |
| VD3 | βCD | 1:2 | In silico | MD | Unraveling mechanism of catalytic isomerization of PreD3 to VD3 | Ferro-Costas 2024 [99] |

**Table S3.** Overview of the vitamin E and CD complexes.

| Vitamin or | CD type | Molar ratio | Preparation method | Analysis method | Aim of study/Application | First author, year of |
|---|---|---|---|---|---|---|

| **vitamin derivative** | | **(vitamin:CD)** | | | | **publication [Article No]** |
|---|---|---|---|---|---|---|
| VE | HPGβCD | 1:1 | Co-precipitation in ethanolic-aqueous solution | $^{1}$H and ROESY NMR, HPLC | Improvement of solubility | Kimura, 2016 [122] |
| VE | βCD | 1.7:1 (mixture of 1:1 and 1:2) | Co-precipitation in aqueous solution | ATR/FTIR, $^{13}$C CP/MAS NMR, TGA, DSC | Enhancement of oxidative stability | Koontz, 2009 [123] |
| VE acetate | LRCD (25-30) | 10:1 | Co-precipitation in ethanolic-aqueous solution | FTIR | Enhancement of solubility | Kuttiyawong, 2015 [145] |
| VE | LRCD (26) | 1:1 | In silico | MD | Enhancement of solubility and stability | Kerdpol, 2021 [124] |
| VE | βCD (chitosan nanoparticles loaded with ternary inclusion complexes MOR/VE/βCD-CS NPs) | 1:1 | Co-precipitation in aqueous-acetone solution and freeze-drying | FTIR, DLS, $^{1}$H NMR, DSC, XRD, AFM, TEM | Increase of hepatoprotective activity against arsenic toxicity | Mondal, 2022 [125] |
| VE | DMβCD | 1:2, 1:3 | Complexation in ethanolic-aqueous solution | ROESY NMR, radical scavenging assays | Enhancement of solubility and antioxidative properties | Ogawa, 2021 [139] |

| | | | | | | |
|---|---|---|---|---|---|---|
| VE | γCD | 1:2, 1:4 | Complexation in ethanolic-aqueous solution | ROESY NMR, radical scavenging assays | Improvement of stabilization | Ogawa, 2021 [140] |
| VE | GluβCD | 1:3 | Co-precipitation in aqueous solution and vacuum drying | DSC, HPLC | Improvement of light stability and water solubility | Okada, 1990 [73] |
| VE | LRCD(26) | 2:1 | Complexation in aqueous solution | HPLC, MD | Enhanced solubility and stability | Sangkhawasi, 2023 [144] |
| VE | βCD HA grafted copolymers crosslinked with DPC | ND | Complexation in ethanolic-aqueous solution and freeze-drying | HPLC, FTIR, $^{1}$H NMR, PXRD, SEM | Increased water solubility | Singh, 2020 [146] |
| VE | βCD | ND | Complexation in ethanolic-aqueous solution and vacuum drying | HPLC, DTG | Prolongation of antioxidative activity of LDPE films | Siró, 2006 [148] |
| VE | βCD loaded in sodium caseinate-coated nanoliposomes | 1:1 | Co-precipitation in ethanolic-aqueous solution and freeze-drying | UV-Vis spectroscopy, FTIR, TEM, PCS, SEM, TGA | Improvement of bioavailability and stability in nutraceuticals and medicines | Souri, 2021 [126] |
| VE, β-TOC, γ-TOC, δ-TOC, VE | βCD, DMβCD, HEβCD | nd | βCD, DMβCD, HEβCD as mobile phase modifiers | HPLC, MEKC | Improvement of separation of VE derivatives | Spencer, 1997 [106] |

| | | | | | | |
|---|---|---|---|---|---|---|
| acetate | | | | | | |
| VE, β-TOC, γ-TOC, δ-TOC, α-TOC3, γ-TOC3, δ-TOC3 | DMβCD | 1:1 | Complexation in aqueous solution | $^{1}H$ and ROESY NMR, EPR | Enhancement of solubility and radical scavenging activity | Sueishi, 2011 [127] |
| VE | βCD | 1:1 | Co-precipitation in ethanolic-aqueous solution, sonication, microwave radiation | FTIR, UV-Vis spectroscopy and FLS | Improved stability, solubility and bioavailability | Jiao, 2010 [128] |
| VE | ODSβCD | 1:1 | Co-precipitation in ethanolic-aqueous solution | SEM, FTIR, $^{13}C$ and NOESY NMR, PXRD, TEM, DLS | Utilization in stabilization of Pickering emulsion | Xi, 2019 [129] |
| VE | βCD amine derivatives | 1:1, 1:2 | Co-precipitation in ethanolic-aqueous solution and freeze-drying | UV-Vis spectroscopy, FLS, $^{1}H$ NMR, DLS, CirD, TEM | Improvement of stability, light and temperature protection | Zhang, 2018 [130] |
| VE | βCD HGNCs | 1:1 | Co-precipitation in aqueous solution | FTIR, DSC, PXRD, SEM, TEM, AFM, CLSM | Development of new controlled release systems with enhanced bioavailability | Eid, 2024 [131] |
| VE | MβCD | 1:1 | Co-precipitation in ethanol solution | ND | Study on protective effects of VE/MβCD on epididymal ram semen during cryopreservation | Benhenia, 2016 [132] |

| | | | | | | |
|---|---|---|---|---|---|---|
| VE | MβCD | 1:1 | Freeze-drying of aqueous solution | ND | Study of protective effects of VE/MβCD on ram sperm during cryopreservation | Benhenia, 2018 [133] |
| VE | OSAβCD | 1:10, 1:12, 1:16, 1:20, 1:25, 1:30 | Co-precipitation from aqueous-isopropanol solution and freeze-drying | FTIR, $^{13}$C CP/MAS NMR, PXRD, SEM, MDoc | Enhancement of physical and oxidative stability of emulsion | Ke, 2020 [143] |
| VE | HPβCD NFs | 1:2, 1:1 | Electrospinning | SEM, FTIR, DSC, PXRD, $^{1}$H NMR, UV-Vis spectroscopy | Prolongation of shelf life and antioxidant activity, enhancement of water solubility and photostability | Celebioglu, 2017 [134] |
| VE | βCD PCL NFs | 1:1 | Electrospinning | PXRD, SEM, TGA | Enhancement of solubility, improved oxidative- and photostability in topical drug delivery | Aytac, 2016 [135] |
| VE | βCD | 1:2, 1:4 | Co-precipitation from aqueous-isopropanol solution and freeze-drying | TGA | Enhancement of thermal stability | Burkeev, 2021 [141] |
| VE | LRCD (9-22) | 1:1, 1,5:1, 2:1, 2.5:1, 3:1 | Co-precipitation in aqueous solution | $^{1}$H NMR, FTIR, TGA, SEM | Improvement of thermostability and antioxidant activity | Cao, 2020 [136] |
| α-TOC3, β-TOC3, γ-TOC3, | PMβCD | ND | PMβCD as mobile phase modifier | HPLC | Improvement of separation of TOC3 derivatives | Drotleff, 1998 [155] |

| | | | | | | |
|---|---|---|---|---|---|---|
| δ-TOC3 | | | | | | |
| TOC3 | γCD | ND | Complexation in aqueous solution and freeze-drying | HPLC, FLS | Enhancement of intestinal absorption in animals | Ikeda, 2010 [147] |
| VE | αCD, βCD, γCD, HPβCD | 1:2,5, 1:5, 1:10 | Evaporation of ethanolic-aqueous solution | FTIR, DSC, PXRD, UV-Vis spectroscopy, MM | Improvement of stability and solubility | Braithwaite, 2017 [20] |
| VE nicotinate, VE acetate | DMβCD | 1:2 | Kneading with small amount of water | PXRD, DTA, HPLC | Improvement of solubility and oral bioavailability | Uekama, 1988 [142] |
| VE acetate | βCD | 1:1, 1:2, 1:3 | Microwave radiation | FTIR, $^{1}$H, $^{13}$C NMR spectroscopy, SEM, MM (PM3) | Enhancement of stability for application in foods | Iskineyeva, 2021 [137] |
| VE | βCD, HPβCD, HPγCD | 1:1, 1:2 | Kneading with small amount of water-methanol solution (1:1, v:v), freeze-drying from aqueous and co-evaporation from water-methanol solution | HPLC, $^{1}$H NMR | Increase of stability | Iaconinoto, 2004 [138] |

**Table S4.** Overview of the vitamin K and CD complexes.

| **Vitamin or vitamin** | **CD type** | **Molar ratio (vitamin:CD)** | **Preparation method** | **Analysis method** | **Aim of study/Application** | **First author, year of publication [Article No]** |
|---|---|---|---|---|---|---|

| | | | | | | |
|---|---|---|---|---|---|---|
| **derivative** | | | | | | |
| VK3 | DMβCD | 1:1 | Co-precipitation from aqueous solution | UV-Vis spectroscopy, $^{1}H/^{13}C$ NMR, CirD | Enhancement of solubility and stability | Szejtli, 1982 [43] |
| VK3 | γCD | 1:1 | Co-precipitation from aqueous solution, co-precipitation from hydroalcoholic solution, kneading with small amount of ethanol | PXRD, DTG, TG, DSC, UV-Vis spectroscopy | Improvement of bioavailability and stability for veterinary use | Lengyel, 1985 [158] |
| VK3 | βCD | 1:1 | Complexation in aqueous solution | FLS | Determination of association constants of complexes and improvement of luminescence detection | Nevado, 2001 [159] |
| VK3 | γCD | 1:1 | Complexing in aqueous solution | PXRD, FTIR UV-Vis spectroscopy, FLS | Studies on inclusion constants and interaction between VK3/γCD complex and herring sperm DNA for antiviral potential | Tang, 2016 [160] |
| VK3 | βCD | 1:1 | In silico | QM calculations (Gaussian) | Thermodynamic studies of complex formation | Petkova, 2023 [40] |
| VK3 | AMCDs | 1:1 | Complexation in aqueous solution | UV-Vis spectroscopy, TEM, DLS | Improvement of solubility and bioavailability | Li, 2019 [166] |
| VK1, VK2 | GluβCD | 1:3 | Co-precipitation in aqueous solution and vacuum drying | DSC, HPLC | Improvement of light stability and water solubility | Okada, 1990 [73] |
| VK3 | αCD, βCD, HPαCD, HPβCD, HPγCD, MβCD | 1:1 | Complexation in aqueous solution | UV-Vis spectroscopy, $^{1}H$ NMR, ITC | Improvement of solubility and analysis of thermodynamic properties | Zielenkiewicz, 2007 [161] |

| | | | | | | |
|---|---|---|---|---|---|---|
| VK3 | βCD, HPβCD, SBEβCD | 1:1 | Complexation in aqueous solution | FLS | Studies on interaction between VK's and CD's molecules in complex and complex structure. Optimization of fluorescence-based detection of VK3 inclusion complexes | Zhenming, 2003 [162] |
| VK3 | βCD, γCD | 1:1, 2:1 | Complexation in water-ethylene glycol solution | PS | Studies on VK3 behavior under irradiation (photodimerization) and its stability | Kuboyama, 1984 [163] |
